# Recurrent requirement for the m^6^A-ECT2/ECT3/ECT4 axis in the control of cell proliferation during plant organogenesis

**DOI:** 10.1242/dev.189134

**Published:** 2020-07-24

**Authors:** Laura Arribas-Hernández, Sara Simonini, Mathias Henning Hansen, Esther Botterweg Paredes, Simon Bressendorff, Yang Dong, Lars Østergaard, Peter Brodersen

**Affiliations:** 1 University of Copenhagen, Department of Biology, Ole Maaløes Vej 5, DK-2200 Copenhagen N, Denmark; 2 John Innes Centre, Colney Lane, Norwich NR4 7UH, UK

**Keywords:** m^6^A, YTH domain, ECT2, ECT3, ECT4, Plant organogenesis

## Abstract

mRNA methylation at the *N6-*position of adenosine (m^6^A) enables multiple layers of post-transcriptional gene control, often via RNA-binding proteins that use a YT521-B homology (YTH) domain for specific m^6^A recognition. In *Arabidopsis*, normal leaf morphogenesis and rate of leaf formation require m^6^A and the YTH-domain proteins ECT2, ECT3 and ECT4. In this study, we show that *ect2/ect3* and *ect2/ect3/ect4* mutants also exhibit slow root and stem growth, slow flower formation, defective directionality of root growth, and aberrant flower and fruit morphology. In all cases, the m^6^A-binding site of ECT proteins is required for *in vivo* function. We also demonstrate that both m^6^A methyltransferase mutants and *ect2/ect3/ect4* exhibit aberrant floral phyllotaxis. Consistent with the delayed organogenesis phenotypes, we observe particularly high expression of *ECT2*, *ECT3* and *ECT4* in rapidly dividing cells of organ primordia. Accordingly, *ect2/ect3/ect4* mutants exhibit decreased rates of cell division in leaf and vascular primordia. Thus, the m^6^A-ECT2/ECT3/ECT4 axis is employed as a recurrent module to stimulate plant organogenesis, at least in part by enabling rapid cellular proliferation.

## Abstract (precis)

**Highlighted Article:** Morphogenesis and growth rate of all plant organs involve the m^6^A-binding proteins ECT2, ECT3 and ECT4.

## INTRODUCTION

In post-embryonic development in plants, organogenesis is the result of activities of the stem cell niches in meristems at the shoot and root apices (Gaillochet and Lohmann, 2015; Pierre-Jerome et al., 2018). Organogenesis involves the distinct steps of initiation of organ primordia from meristematic cells, separation of primordia from meristems via boundary formation, and cellular proliferation and expansion coupled with differentiation (Bar and Ori, 2014; Du et al., 2018; Thomson and Wellmer, 2019; Wachsman et al., 2015). Key molecular principles governing these processes are signaling by the hormones auxin and cytokinin (Schaller et al., 2015), and establishment of mutually exclusive transcriptional programs via specific expression of antagonistic transcription factors (Drapek et al., 2017). In the main root meristem, the normally non-dividing quiescent center (QC) is defined by expression of the transcription factor WUSCHEL-LIKE HOMEOBOX5 (WOX5). An auxin maximum marks the *WOX5*-expressing QC cells that signal stem cell identity to immediately surrounding cells (Blilou et al., 2005; Forzani et al., 2014; Petersson et al., 2009; Sabatini et al., 1999; van den Berg et al., 1997), while cytokinin drives the transition to differentiation of the root stem cells (Dello Ioio et al., 2007) (see Drisch and Stahl, 2015 for a review). Conversely, at the shoot apical meristem (SAM), high cytokinin levels are present in the organizing center (OC), defined by expression of the founding WOX family member *WUSCHEL* (*WUS*). Stem cell identity in the adjacent central zone is specified by the non-cell autonomous action of WUS, and division of those stem cells is promoted by cytokinin (Chickarmane et al., 2012; Werner et al., 2003). Auxin, on the other hand, drives stem cell differentiation in the SAM, as lateral organ primordia with high cell division rates initiate from sites of auxin maxima at the periphery of the meristem (see Byrne, 2012 for a review). This process requires the repression of the KNOTTED-LIKE HOMEOBOX (KNOX) family of meristematic transcription factors that includes SHOOT-MERISTEMLESS (STM) (Hay and Tsiantis, 2010; Long et al., 1996). As the cells in that region engage in proliferation, the emerging primordium becomes an auxin sink, and depletion of auxin from the surrounding area prevents formation of adjacent primordia (Bartlett and Thompson, 2014; Benková et al., 2003; Heisler et al., 2005; Reinhardt et al., 2003). A number of microRNAs (miRNAs) has also been shown to reinforce robustness of the gene regulatory circuits specifying root, leaf and flower formation through regulation of the key transcription factors (Rubio-Somoza and Weigel, 2011). Nonetheless, the involvement of other mechanisms of post-transcriptional gene control in plant organogenesis remains poorly investigated.

Methylation of adenosine at the *N6*-position (m^6^A) in mRNA has recently emerged as a widespread mechanism of gene regulation (Zaccara et al., 2019). In eukaryotes, m^6^A is installed co-transcriptionally by a conserved, multi-subunit complex, the catalytic core of which consists of two methyltransferase-like proteins, METTL3 and METTL14 (Bokar et al., 1994, 1997; Liu et al., 2014), in plants called MTA and MTB, respectively (Růžička et al., 2017; Zhong et al., 2008). This heterodimer associates with additional proteins that are also required for m^6^A methyltransferase activity *in vivo* (Balacco and Soller, 2019). Their orthologs in plants include the splicing factor FKBP12 INTERACTING PROTEIN of 37 kDa, FIP37 [WTAP/Fl(2)d in metazoans] (Shen et al., 2016; Vespa et al., 2004; Zhong et al., 2008), the large protein of unknown biochemical function VIRILIZER (VIR) and the putative ubiquitin ligase HAKAI (Růžička et al., 2017). m^6^A is required for embryonic development beyond the globular stage in plants (Růžička et al., 2017; Zhong et al., 2008) and is key to post-embryonic development, as hypomorphic *vir-1* mutants or plants post-embryonically depleted of MTA exhibit stunted growth, severe developmental defects and a 75-90% reduction in m^6^A/A ratio compared with wild type (Bodi et al., 2012; Růžička et al., 2017). Similarly, post-embryonic depletion of FIP37 results in strongly delayed and defective leaf formation: the SAM overproliferates and fails to produce leaf primordia at its flanks, or does so with a strong delay compared with wild type (Shen et al., 2016).

Many effects of m^6^A are mediated by RNA-binding proteins harboring a YT521-B homology (YTH) domain (Hartmann et al., 1999; Imai et al., 1998; Stoilov et al., 2002; Zhang et al., 2010) that is specialized for m^6^A recognition. The YTH domain contains a hydrophobic pocket consisting of highly conserved aromatic amino acid residues (the ‘aromatic cage’) that accommodate the *N6*-methyl group and thereby increase the affinity for m^6^A-containing RNA by 10- to 20-fold over unmethylated RNA of the same sequence (Li et al., 2014b; Luo and Tong, 2014; Theler et al., 2014; Xu et al., 2014; Zhu et al., 2014). The phylogeny of YTH domains defines two major classes, YTHDF and YTHDC, that may be found in several different proteins (Zaccara et al., 2019). YTHDF proteins are typically cytoplasmic and, in mammals, the molecular effects of the three family members (YTHDF1-3) can either be to accelerate the decay of m^6^A-containing mRNAs or to enhance their translation (Du et al., 2016; Kennedy et al., 2016; Li et al., 2017; Park et al., 2019; Sheng et al., 2020; Shi et al., 2017; Wang et al., 2014, 2015) (see Patil et al., 2018 for a review). The biological relevance of YTHDF2-mediated mRNA decay has been proposed in several germline and somatic cell differentiation-related processes (Ivanova et al., 2017; Li et al., 2018; Zhang et al., 2017; Zhao et al., 2017), while YTHDF1-mediated translational activation is required for some neuronal functions (Shi et al., 2018; Weng et al., 2018).

Plant genomes encode an expanded set of YTHDF proteins, referred to as EVOLUTIONARILY CONSERVED C-TERMINUS (ECT), of which 11 are found in *Arabidopsis* (Li et al., 2014a; Scutenaire et al., 2018). The YTH domains of ECT1-11 contain all amino acid residues crucial for m^6^A binding (Fray and Simpson, 2015) and m^6^A-binding activity has been directly shown for ECT2 (Wei et al., 2018). Furthermore, the m^6^A-binding capacity of ECT2 and ECT3 and its *in vivo* relevance are inferred from failure of m^6^A pocket-disrupting mutants to restore the phenotypes of their corresponding knockout mutants (Arribas-Hernández et al., 2018; Scutenaire et al., 2018; Wei et al., 2018). In contrast, the downstream molecular effects of plant YTHDF proteins remain unclear (Arribas-Hernández and Brodersen, 2020).

We recently found that the three YTHDF proteins, ECT2, ECT3 and ECT4, perform genetically redundant functions in leaf formation (Arribas-Hernández et al., 2018): *ect2/ect3* double mutants complete post-embryonic leaf formation with a substantial delay compared with wild type, a phenotype that is exacerbated by additional mutation of *ECT4*. The leaves of *ect2/ect3/ect4* triple mutants have serrated edges and a triangular (deltoid) shape that strongly resembles that of *mta* knockdown plants (Arribas-Hernández and Brodersen, 2020; Shen et al., 2016). *ect2/ect3* mutants also exhibit defective control of branching of unicellular epidermal hairs (trichomes), and weaker trichome branching defects can also be observed in *ect3* (Arribas-Hernández et al., 2018) and *ect2* (Arribas-Hernández et al., 2018; Scutenaire et al., 2018; Wei et al., 2018) single mutants. It remains unclear, however, whether the important functions of ECT2, ECT3 and ECT4 in leaf development rely on functions within the SAM or in developing leaf primordia, or both, and whether the involvement of the m^6^A-ECT2/ECT3/ECT4 module is specific to leaf formation or general to plant organogenesis. Similarly, the basis for the defects in embryogenesis and morphogenesis of roots, shoots and flowers of m^6^A-deficient mutants (Bodi et al., 2012; Růžička et al., 2017; Shen et al., 2016; Vespa et al., 2004; Zhong et al., 2008) remains ill defined. Most fundamentally, the issue of whether these important biological effects involve ECT proteins is still unresolved.

In this study, we show that the m^6^A-ECT2/ECT3/ECT4 module is necessary for correct root, flower and fruit formation. ECT2, ECT3 and ECT4 are highly expressed in rapidly dividing cells of organ primordia and only weakly expressed in peripheral meristematic cells, with little or no expression detectable in organizing or quiescent centers of inflorescence and root apical meristems. Consistent with these expression patterns, we observe slower growth of leaf primordia due to reduced rate of cell proliferation in *ect2/ect3/ect4* triple mutants, but no clear delay in initiation of leaf primordia. Furthermore, the size of both vegetative and inflorescence meristems in *ect2/ect3/ect4* triple mutants appears normal. Together, these observations establish that the m^6^A-ECT2/ECT3/ECT4 module is generally required for plant organogenesis, presumably via stimulation of cell proliferation in organ primordia.

## RESULTS

### Leaf primordia of *ect2*/*ect3*/*ect4* mutants exhibit reduced cellular proliferation, but not delayed initiation

**Fig. 1.**
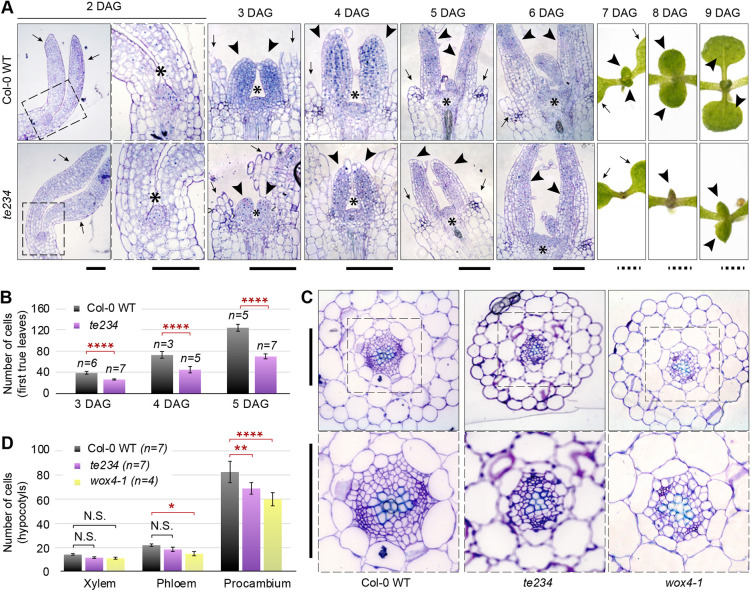
**Delayed growth in *ect2/ect3/ect4* is explained by low cell division rate in leaf primordia.** (A) Toluidine blue-stained longitudinal histological sections (days 2-6) or photographs (days 7-9) show the development of the first true leaves. Outlined areas are magnified in the adjacent images. Arrows, cotyledons (broken in 3-6 DAG samples); arrowheads, first true leaves; asterisks, shoot apical meristem (SAM). (B) Quantification (data are mean±s.e.m.) of the number of epidermal cells in longitudinal sections of the two first true leaves. Number of individuals measured (*n*) is indicated; ****P<0.0001, post-hoc pairwise comparison. N.S., not significant. (C) Toluidine blue-stained histological cross-sections of hypocotyls of 10-day-old seedlings. Areas outlined are magnified below to show the cells inside vascular bundles. (D) Quantification (data are mean±s.e.m.) of the number of vascular cells. Number of individuals measured (*n*) is indicated; *P<0.05, **P<0.01, ****P<0.0001, post-hoc pairwise comparison. Scale bars: 100 μm in sections; 1 mm in whole leaves.

We first analyzed shoot apices of *ect2-1/ect3-1/ect4-2* seedlings (referred to here as *te234* for *triple ect234*, see Table 1 for abbreviations of all *ect* mutant allele combinations) to assess whether the SAM was visibly affected, and whether the initiation of leaf primordia was delayed. We observed no significant difference in SAM size between *te234* and wild type from day 2 to 6 post-germination (Fig. 1A; see Fig. S1). Importantly, we could not detect any difference in the timing of emergence of leaf primordia, as it occurred between 2 and 3 days after germination (DAG) in both cases (Fig. 1A). On the contrary, counts of epidermal cells in leaf primordia as a function of time revealed that the estimated doubling time was significantly longer in *te234* mutants than in wild type [Fig. 1B; t_2_(Col-0)=26.1 h, t_2_(*te234*)=34.5 h; *P*<0.001, see Materials and Methods], although no obvious difference in cell size was apparent at that stage. These analyses suggest that reduced growth rate of leaf primordia as a consequence of reduced cellular proliferation is the primary cause of the delayed leaf emergence in *ect2/ect3/ect4* mutants. This is in contrast to *fip37* knockdown plants in which meristems overproliferate and form leaf primordia with a significant delay (Shen et al., 2016). Thus, m^6^A appears to affect leaf formation at least at two different levels: (1) initiation of leaf primordia via mechanisms that do not depend on ECT2, ECT3 and ECT4; and (2) growth of leaf primordia via mechanisms that involve rapid cellular proliferation and that require ECT2, ECT3 and ECT4.

**Table 1.**
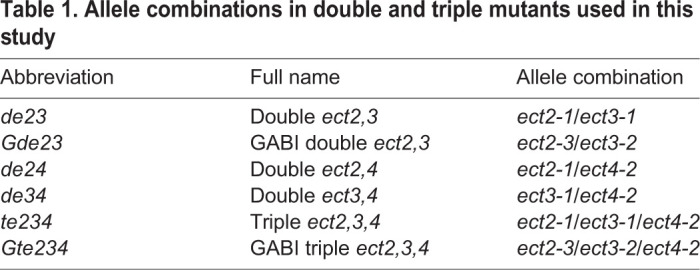
**Allele combinations in double and triple mutants used in this study**

### Cellular proliferation of vascular stem cells is also reduced in *ect2*/*ect3*/*ect4* mutants

To assess whether the low cell division rate also occurs in other developmental contexts, we examined vascular stem cells in hypocotyls. Vascular stem cells, or procambium, continuously proliferate as they self-maintain and give rise to the mature vascular tissues, xylem and phloem. Procambial proliferation requires the transcription factor WUSCHEL-related HOMEOBOX4 (WOX4) (Hirakawa et al., 2010; Ji et al., 2010; Suer et al., 2011). We therefore analyzed cross-sections of wild-type and *te234* hypocotyls, and included the *wox4-1* knockout mutant (Hirakawa et al., 2010) as a control for procambial proliferation defects. Compared with wild type, *te234* mutants showed a significant reduction in the number of vascular meristematic cells, but no significant differences in the number of mature xylem and phloem cells (Fig. 1C,D). This result suggests that ECT2, ECT3 and ECT4 also potentiate cell division in vascular stem cells, and hence points towards a general role of ECT2, ECT3 and ECT4 in promoting cell proliferation.

### Arrest of growth in leaves of *ect2*/*ect3*/*ect4* mutants is delayed

**Fig. 2.**
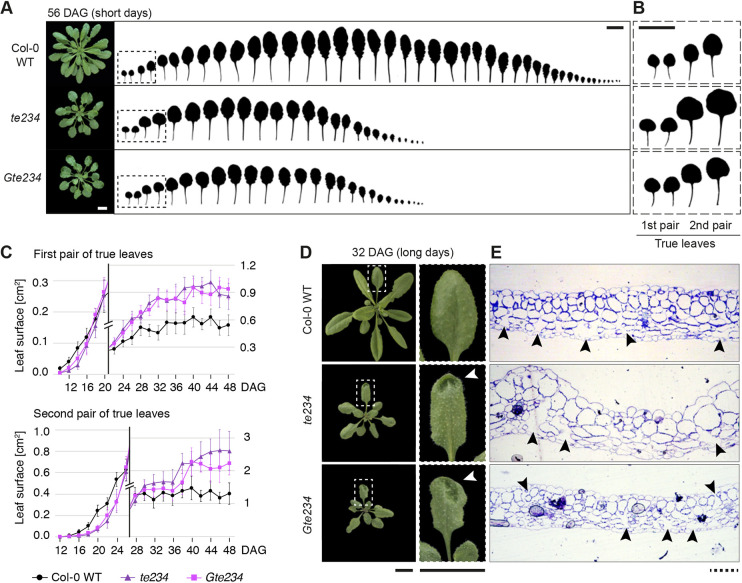
**Leaf formation and size are defective in *ect2/ect3/ect4* mutants.** (A) Rosettes and detached leaves of plants grown in short days for 56 days. (B) Magnification of the outlined areas in A. (C) Area of each of the first and second pairs of true leaves over time, grown in short days (data are mean±s.d., *n*=16-20 leaves). A double scale in cm^2^, indicated on the left and right sides of both graphs, is used to show in detail both early and late stages of growth. (D) Photographs of wild type and *ect2/ect3/ect4* mutants grown in long days for 32 days. Leaves with concavities on the surface (white arrowheads) are outlined and magnified in the right panels. A wild-type leaf is also magnified for comparison. (E) Toluidine blue-stained transverse histological sections through concavities in leaves of *ect2/ect3/ect4* mutants and comparable areas (leaf size and position on the lamina) of Col-0 wild-type leaves. Black arrowheads indicate intercellular spaces. Scale bars: 2 cm in whole leaves; 100 μm in sections.

Our previous analyses of leaf formation suggested that *te234* mutants may display two defects compared with wild type (Arribas-Hernández et al., 2018): (1) slower pace of leaf formation throughout rosette development; and (2) larger final leaf size despite later emergence. To rigorously document the latter phenomenon, we grew two independent allele combinations of *ect2/ect3/ect4* under short-day conditions to prevent floral transition, and measured the area of juvenile leaves throughout the growth period (Fig. 2A-C). This quantification clearly demonstrated the two distinct defects suggested by our previous observations, and exposed the fact that leaf growth remains active for roughly two weeks longer in the mutants than in wild type, such that the final size of the first two pairs of leaves is 1.5- to 2-fold greater (*P*<0.0001 at 48 DAG for pairwise comparisons, with no significant differences between the two mutant alleles, see Materials and Methods) (Fig. 2B,C). Thus, both initial leaf growth rates and the timing of growth arrest are affected in *ect2/ect3/ect4* mutants.

### Leaf blades in the *ect2*/*ect3*/*ect4* mutant exhibit deformities

We also noticed that irregular concavities in the leaf surface frequently occur in *ect2/ect3/ect4* mutants (Fig. 2D). The formation of the flat leaf disc requires coordination of cell division rates and anisotropic growth along proximodistal and mediolateral axes (Fox et al., 2018). Defects in such coordination may cause mechanical stretch, and thereby give rise to surface irregularities. Indeed, transverse histological sections through such concavities showed irregular numbers of cell layers and cell sizes, and disorganized disposition of cell types in *ect2/ect3/ect4* compared with wild type (Fig. 2E). In particular, the intercellular spaces that occur exclusively on the abaxial side of the blade in wild-type plants were of more irregular sizes and occasionally appeared on the adaxial size of the mutant leaves, perhaps suggesting defects in leaf polarity (Fig. 2E). We conclude that leaf growth proceeds with multiple defects in rate, timing, coordination and patterning upon loss of the m^6^A-ECT2/ECT3/ECT4 axis.

### ECT2, ECT3 and ECT4 are highly expressed at the root apex and throughout lateral root formation

**Fig. 3.**
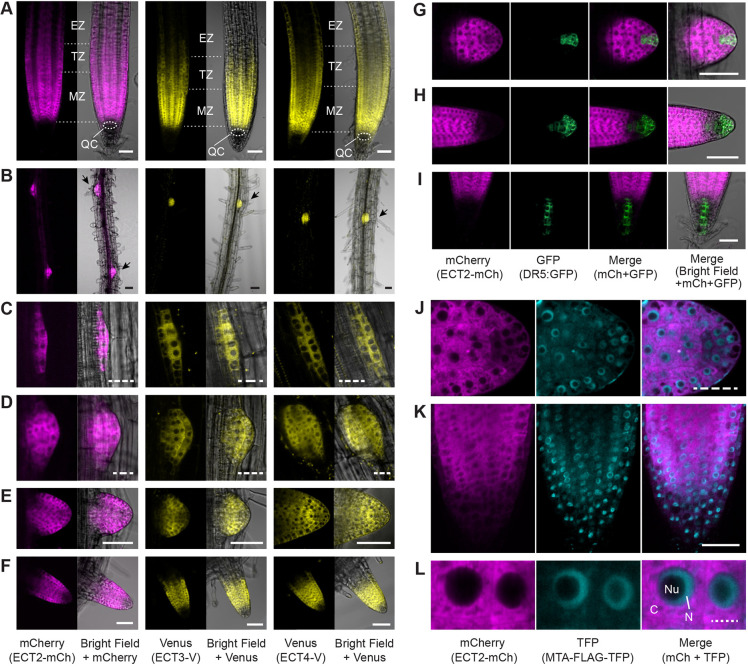
**Expression of *ECT2*, *ECT3* and *ECT4* in root tips and lateral root primordia.** Confocal fluorescence microscopy images of roots of 6- to 9-day-old seedlings expressing *ECT2-mCherry*, *ECT3-Venus* or *ECT4-Venus*. (A) Main root tips. QC, quiescent center; MZ, meristematic zone; TZ, transition zone; EZ, elongation zone (Wachsman et al., 2015). (B) Main roots with budding lateral root primordia (black arrows). (C-E) Lateral root primordia at stages II (C), V-VI (D) and VII-VIII (E) (Péret et al., 2009). (F) Tips of emerging lateral roots. (G-I) Lateral root primordium (G), and lateral (H) and main (I) root tips co-expressing *ECT2-mCherry* and *DR5:GFP*. (J-L) Lateral root primordium (J) and main root tip (K,L) co-expressing *ECT2-mCherry* and *MTA-FLAG-TFP*. C, cytoplasm; N, nucleoplasm; Nu, nucleolus. Scale bars: 50 μm in A,B,E-I,K; 25 μm in C,D,J; 5 μm in L.

We next studied the possible relevance of ECT2, ECT3 and ECT4 in root formation, as mutants deficient in m^6^A deposition display impaired root growth and gravitropism (Růžička et al., 2017), and m^6^A writer components are highly expressed in root meristems and/or lateral root primordia (Růžička et al., 2017; Zhong et al., 2008). Our analyses started with a thorough examination of *ECT2*, *ECT3* and *ECT4* expression patterns using stable lines of *ECT2-mCherry*, *ECT3-Venus* and *ECT4-Venus* fusions that showed strong expression in root tips (Fig. 3A), as previously reported (Arribas-Hernández et al., 2018). Along the rest of the root, expression of all three proteins was highest at the sites of lateral root formation (Fig. 3B) with much weaker fluorescence seen in the vasculature, in particular for ECT2. More detailed analyses revealed high expression of ECT2, ECT3 and ECT4 in sites of lateral root initiation after the first periclinal division at stage II (Péret et al., 2009) (Fig. 3C). The signal remained high in all cells throughout the early stages of lateral root development (Fig. 3D), but was ultimately restricted to the proliferative area of newly formed lateral roots (Fig. 3E,F) in a pattern identical to that of the main root tips (Fig. 3A). To clearly visualize the exclusion of ECT2 expression from the QC, we introduced the auxin-responsive *DR5:GFP* reporter, with specific expression in cells of the QC and of the columella (Benková et al., 2003; Ulmasov et al., 1997), into *ECT2-mCherry* lines. This analysis confirmed that *ECT2* is not expressed in the QC itself, but in the adjacent cell division zone (Fig. 3G-I). It also revealed that although *ECT2*, *ECT3* and *ECT4* are highly expressed in cells experiencing an auxin maximum during early stages of lateral root formation, the expression of at least *ECT2* is specifically excluded from the newly formed auxin maximum at emerging lateral root tips (Fig. 3G). Overall, we conclude that expression of *ECT2*, *ECT3* and *ECT4* in the root is particularly strong in proliferating cells undergoing differentiation.

### The expression domain of MTA, but not of ECT2, ECT3 and ECT4, includes the QC

The exclusion of *ECT2*, *ECT3* and *ECT4* from the QC raises the issue of whether the *ECT2*, *ECT3* and *ECT4* expression pattern reflects the full range of cells in which m^6^A is used to control gene expression. To address this, we generated a C-terminal fusion of MTA to the turquoise fluorescent protein (TFP; *MTA-FLAG-TFP*) under the control of the *MTA* native promoter, and demonstrated its functionality by complementation of the embryonically lethal *mta-2* knockout mutants (see Fig. S2). Confocal microscopy of lines expressing both *MTA-FLAG-TFP* and *ECT2-mCherry* demonstrated that, although *MTA-FLAG-TFP* and *ECT2-mCherry* are co-expressed in the division zone of the root meristem, only signal from MTA-FLAG-TFP is visible in the QC (Fig. 3J,K). Thus, although ECT2, ECT3 and ECT4 are key effectors of the m^6^A pathway in the division zone, other effectors, potentially other ECT proteins, are likely to mediate m^6^A functions in the QC.

### Distinct subcellular localization of MTA and ECT2

We also used the *MTA-FLAG-TFP*/*ECT2-mCherry* co-expressing lines to compare the subcellular localization of the two proteins. Whereas MTA-FLAG-TFP was nucleoplasmic, ECT2-mCherry was predominantly cytoplasmic and did not overlap with MTA-FLAG-TFP (Fig. 3L). On the contrary, an area around the nucleus from which both proteins were excluded, presumably containing the nuclear envelope, was clearly visible in the merged images (Fig. 3L). These observations are consistent with a compartmentalized m^6^A-YTHDF pathway in which the m^6^A mark is written in the nucleus and read by ECT2 in the cytoplasm. Nonetheless, the resolution employed here does not allow us to totally exclude the presence of ECT2 in the inner nuclear periphery, as was previously suggested (Scutenaire et al., 2018; Wei et al., 2018).

### ECT2, ECT3 and ECT4 are required for normal rate and directionality of primary root growth

**Fig. 4.**
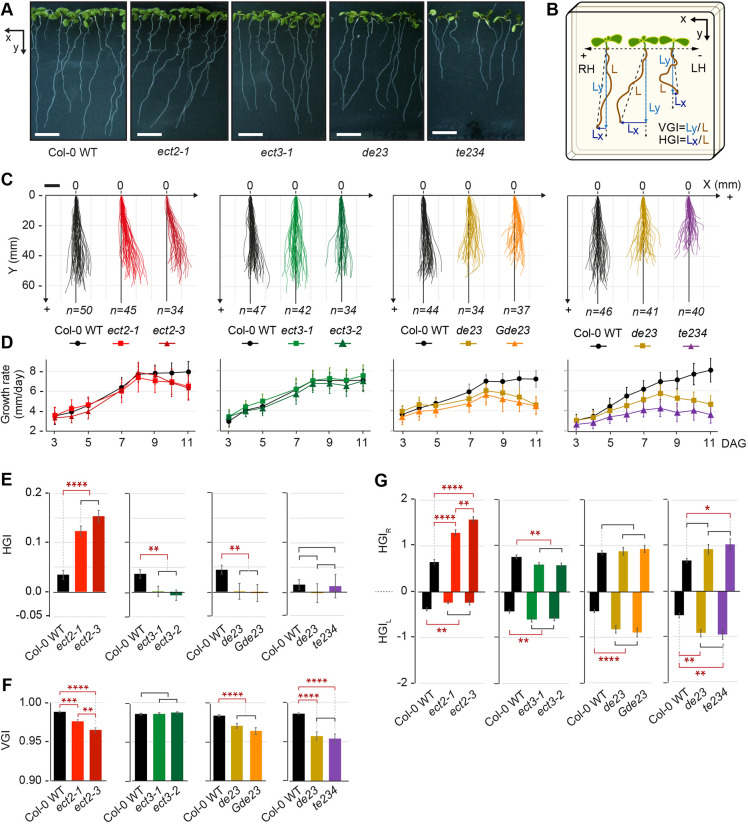
**Defects in root growth rate and directionality in single and multiple *ect2/ect3/ect4* mutants.** (A) Photographs of 9-day-old seedlings grown vertically. The orientation of the *x*/*y* axes is indicated (frontside picture). (B) Illustration of the growth conditions for the photographs in A, and the calculation of vertical and horizontal growth indices (VGI and HGI). L, length; RH, right-handedness; LH, left-handedness. (C-G) Characterization of roots of *ect2*, *ect3*, *ect2/ect3* and *ect2/ect3/ect4* mutants compared with wild type: (C) representation of the growth pattern in a two-dimensional *x/y* space as observed from the backside of the plate; (D) growth rate in mm/day (data are mean±s.d.); (E,F) VGI and HGI at 11 DAG (data are mean±s.e.m.); (G) cumulative left and right horizontal growth indices (HGI_L_/HGI_R_) representing the sum of daily HGIs from days 2-3 to 10-11 (data are mean±s.e.m.). *P<0.05, **P<0.01, ***P<0.001, ****P<0.0001, one-way ANOVA followed by post-hoc pairwise comparison. When no significant difference (black brackets) was found between alleles of the same gene, their values were combined for a more robust comparison to wild type (shown). Scale bars: 10 mm.

We next analyzed whether ECT2, ECT3 and ECT4 are functionally relevant for root growth. Initial observations of root growth in single, double and triple mutants suggested that *ect2/ect3* double and, in particular, *ect2/ect3/ect4* triple mutants exhibited slower root growth and more agravitropic behavior than wild type (Fig. 4A; see Fig. S3A,B), as do plants with reduced m^6^A levels (Růžička et al., 2017). In addition, *ect2* single mutants exhibited exacerbated right slanting of root growth compared with the weak right slanting of Col-0 wild type (Grabov et al., 2005; Migliaccio and Piconese, 2001) (Fig. 4A). To quantify these phenotypes, we recorded the position of root tips every 24 h from 3 to 11 DAG to generate data for three informative morphometric characterizations: (1) a graphic description of root phenotypes as an overlay of the individual root growth trajectories; (2) root length and growth rate as a function of time; and (3) vertical and horizontal growth indices (VGI and HGI, respectively) (Grabov et al., 2005) (Fig. 4B-F; see Fig. S3C). To better describe slanting (Ferrari et al., 2000) and meandrous (agravitropic) growth, we also calculated partial HGI indices for every daily increment in growth, categorized them into left (−) and right (+) classes, and summed them to obtain cumulative left and right horizontal growth indices (HGI_L_ and HGI_R_) (Fig. 4G). In this way, differences between genotypes could be quantified and the statistical significances of such differences assessed.

The quantitative analyses confirmed that roots of two independent *ect2* single mutants display exacerbated right slanting (Fig. 4C), as revealed by a significantly higher positive HGI compared with Col-0 wild type (Fig. 4E). Accordingly, *ect2* mutants had a higher |HGI_R_|, but lower |HGI_L_| and VGI compared with wild type (Fig. 4F,G). Strikingly, we observed the opposite tendency in *ect3* single mutants. Roots of two different *ect3* mutants had negligible slanting as shown by near-zero HGI scores compared with the highly reproducible positive HGI (∼0.015-0.045) in Col-0 wild type (Fig. 4E). Correspondingly, *ect3* mutants had a significantly lower |HGI_R_|, but higher |HGI_L_| than wild type (Fig. 4G), thereby producing VGI scores similar to those of wild type (Fig. 4F).

In contrast, roots of two different allele combinations of *ect2/ect3* double mutants exhibited meandrous growth rather than slanting (Fig. 4C): the VGI was low (comparable with that of *ect2*), which is indicative of non-vertical growth, but the HGI was near-zero (comparable to that of *ect3*) (Fig. 4E,F). Accordingly, |HGI_L_| and |HGI_R_| values were alike, but in this case both were higher than those of *ect3* (Fig. 4G). Most importantly, *ect2/ect3* seedlings had clearly reduced root growth rates (Fig. 4D; *P*<0.0001 for pairwise comparison between wild type and both *ect2/ect3* double mutants at 7-11 DAG). Finally, the single mutation of *ect4* did not produce significant differences in root growth rate or directionality, and *ect2/ect4* resembled *ect2*, whereas *ect3/ect4* resembled *ect3* (see Fig. S4). However, the slow root growth of *de23* seedlings was exacerbated by mutation of *ECT4* (*P*<0.0001 for pairwise comparison between *de23* and *te234* during 6-11 DAG) (Fig. 4C,D). We conclude that, similar to their role in leaf formation (Arribas-Hernández et al., 2018), ECT2, ECT3 and ECT4 act redundantly to promote the rate of root growth, consistent with their high expression in the division zone of root meristems. However, specific, and even opposite, effects of ECT2 and ECT3 can be detected in root growth directionality, pointing to the existence of either specific mRNA targets of ECT2 and ECT3, or to differences in their mode of mRNA regulation.

### Binding to m^6^A is required for the function of ECT2 and ECT3 in root morphogenesis

**Fig. 5.**
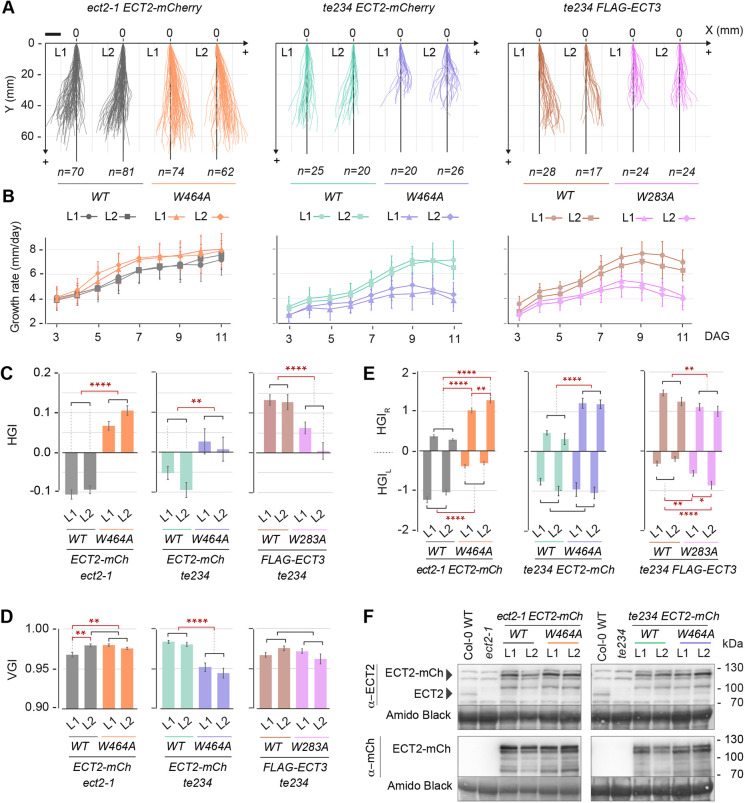
**Normal root growth and directionality require intact m^6^A-binding sites in ECT2 and ECT3.** (A-E) Characterization of roots of the indicated stable transgenic lines. (A) representation of the growth pattern in a two-dimensional *x/*y space as observed from the backside of the plate; (B) growth rate in mm/day (data are mean±s.d.); (C,D) VGI and HGI at 11 DAG (data are mean±s.e.m.); (E) cumulative left and right horizontal growth indices (HGI_L_/HGI_R_) representing the sum of daily HGIs from days 2-3 to 10-11 (data are mean±s.e.m.). *P<0.05, **P<0.01, ****P<0.0001, one-way ANOVA followed by post-hoc pairwise comparison. (F) Western blots of total protein extracts of vertically grown 10-day-old seedlings probed with antibodies recognizing ECT2 or mCherry.

To test whether the m^6^A-binding activity of ECT2 is involved in root slanting, we characterized root growth of *ect2-1* mutants expressing either *ECT2-mCherry* or its aromatic cage mutant, *ECT2^W464A^-mCherry*, under the control of the *ECT2* native promoter (Arribas-Hernández et al., 2018). Interestingly, expression of the wild-type transgene, but not the m^6^A-binding deficient mutant, not only rescued the enhanced right slanting of *ect2-1*, but also inverted the root growth directionality to a left slanting, contrary to the natural tendency of the Col-0 ecotype (Grabov et al., 2005; Migliaccio and Piconese, 2001), as seen by negative HGI values and |HGI_L_|>|HGI_R_| (Fig. 5A-E). As ECT2-mCherry levels in the transgenic lines exceed endogenous ECT2 levels (Fig. 5F), we conclude that root growth directionality exhibits exquisite ECT2 dose dependence: exacerbated right slanting is seen in *ect2* mutants, while even weak ECT2 overexpression causes left slanting.

Next, we tested whether the m^6^A-binding activities of both ECT2 and ECT3 are necessary for the correct growth rate of roots, using transgenic lines expressing either *ECT2-mCherry*, *FLAG-ECT3* or their corresponding aromatic cage mutants, *ECT2^W464A^-mCherry* and *FLAG-ECT3^W283A^*, but this time in the *te234* mutant background (Arribas-Hernández et al., 2018). These experiments showed that expression of the wild-type *ECT2* and *ECT3* transgenes largely rescued the growth defects observed in roots of *te234* mutants, while the m^6^A-binding-deficient versions failed to do so (Fig. 5A,B). Also in this case, lines slightly overexpressing *ECT2-mCherry* showed left slanting (Fig. 5A,C,E-F), while *te234* plants partially complemented by *FLAG-ECT3* recapitulated the magnitude of right slanting seen in *de24* mutants (compare Fig. 5 with Fig. S4). Thus, primary root growth, including both rate and directionality, requires the m^6^A-ECT2/ECT3/ECT4 module.

### ECT2, ECT3 and ECT4 are highly expressed in floral primordia

**Fig. 6.**
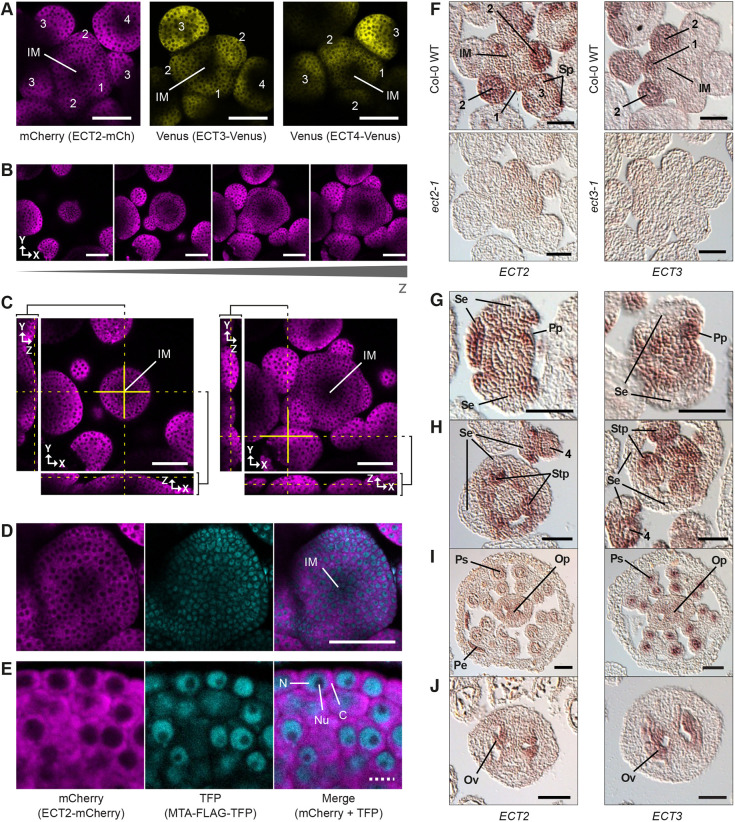
**Expression of *ECT2*, *ECT3* and *ECT4* in inflorescence meristems and floral primordia.** (A) Confocal fluorescence microscopy images of primary shoot inflorescences expressing *ECT2-mCherry, ECT3-Venus* or *ECT4-Venus*. (B) Four representative focal planes of increasing depth (*z* is the growth axis) from a *z*-section stack of an inflorescence meristem (IM) expressing *ECT2-mCherry*. The remaining sections and a 3D projection are shown in Fig. S5A,B. (C) Orthogonal views of two focal planes of the *z*-stack in B. Left, views through the center of the IM; right, views through a young flower primordium. (D,E) IM (D) and surrounding early floral primordia (D,E) of plants co-expressing *ECT2-mCherry* and *MTA-FLAG-TFP*. C, cytoplasm; N, nucleoplasm; Nu, nucleolus. (F-J) *ECT2* and *ECT3* mRNA detected by *in situ* hybridization of tissue cross-sections of Col-0 wild type, *ect2-1* or *ect3-1* inflorescences. Hybridization to antisense probes (Fig. S5C) is revealed by red color. (F) IMs with young (stage 1-3) floral primordia. Dashed lines mark the boundaries of assemblage of two consecutive sections (the entire sections are shown in Fig. S6A,B). (G-J) Hybridization on sections of Col-0 wild-type floral primordia at later stages. See comparable sections of *ect2-1* and *ect3-1* controls and sections of additional tissues in Figs S6, S7. Numbers refer to stages of floral development (Smyth et al., 1990). IM, inflorescence meristem. Sp, sepal primordium; Se, sepal; Pp, petal primordium; Pe, petal; Stp, stamen primordium; Ta, tapetum; Op, ovule primordium. Scale bars: 50 μm in A-D,F-J; 5 μm in E.

We next examined reproductive tissues. Expression of fluorescent fusions of *ECT2*, *ECT3* and *ECT4* was detected throughout the inflorescence meristematic area, but the signal was higher in cells of young floral primordia than in the inflorescence meristem (IM) (Fig. 6A). This difference was not due to attenuation of the signal with tissue depth, because orthogonal views of *z*-stacks of meristems revealed that floral primordia had higher ECT2-mCherry fluorescence intensities than the IM at comparable depths (Fig. 6B,C; Fig. S5A,B). Furthermore, when we examined transgenic lines co-expressing *MTA-FLAG-TFP* and *ECT2-mCherry*, we observed nuclear TFP signal throughout the meristem, including in the central zone devoid of ECT2-mCherry signal (Fig. 6D), reminiscent of the pattern observed in root tips. Again, the subcellular localizations of MTA-FLAG-TFP and ECT2-mCherry were complementary, with MTA-FLAG-TFP being nucleoplasmic and ECT2-mCherry being mainly cytoplasmic (Fig. 6E), as observed in roots.

To validate the expression pattern displayed by the fluorescent fusion proteins and to characterize the expression of *ECT2* and *ECT3* in floral organ primordia, we performed RNA *in situ* hybridization with probes specific for *ECT2* and *ECT3* mRNAs (Fig. 6F; see Figs S5C, S6 and S7). Expression of *ECT2/3* mRNA was highest in young floral primordia (stages 1-2; Smyth et al., 1990), while weaker signal was observed in the IM (Fig. 6F). This result is in agreement with the fluorescence microscopy, and therefore strongly supports accurate reflection of the endogenous expression pattern by our fluorescent reporters. At later stages, the signal was located at sepal, petal and stamen primordia (Fig. 6F-H), but only at the edges of developing sepals and petals, and in gametes of more mature flower buds (Fig. 6G-J; see Figs S6H and S7G). In summary, *ECT2* and *ECT3* are mainly expressed in young floral and floral organ primordia undergoing cell proliferation and differentiation. The IM itself shows less expression, mostly in the peripheral zones and little expression is seen in more developed organs. Thus, also in flower formation, ECT2 and ECT3 (and possibly ECT4) exert their functions mainly in rapidly dividing cells.

### *ect2*/*ect3*/*ect4* and m^6^A writer mutants exhibit defective floral phyllotaxis

**Fig. 7.**
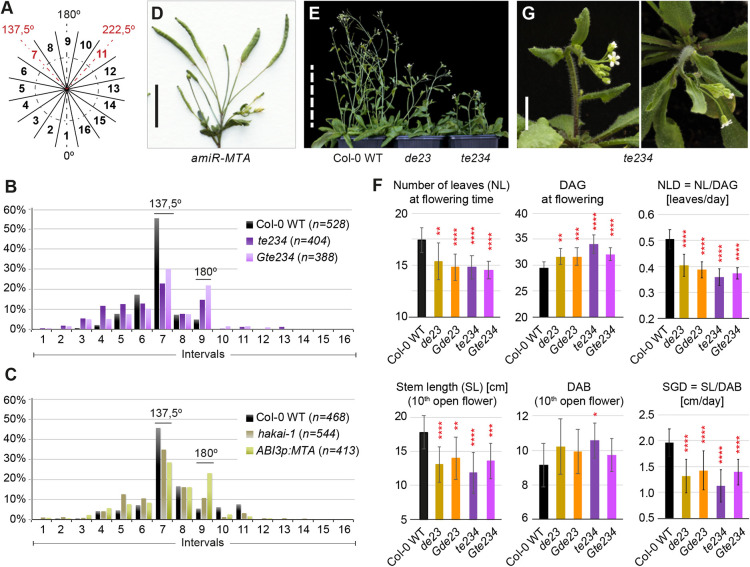
**Defective timing, growth rate and morphogenesis of stems in *ect2/ect3/ect4* mutants.** (A-C) Phyllotaxis analysis showing the distribution of divergence angles between two successive flowers. The frequency distribution across intervals 1-16 (A) for *n* measurements of the indicated genotypes is shown (B,C). (D) Main stem of a plant expressing *amiR-MTA* (Shen et al., 2016). (E) 40-day-old plants grown in long days. (F) Histograms (data are mean±s.d.) showing number of leaves (NL) and DAG at flowering time, the rate of leaf formation during that time (number of leaves per day, NLD), the time (days after bolting, DAB) and stem length [SL (cm)] at the time at which the 10th flower opens, and the rate of stem growth during that time [stem growth per day, SGD (cm/day)] (*n=*17-20, see Materials and Methods for details). *P<0.05, **P<0.01, ***P<0.001, ****P<0.0001, relative to wild type, obtained by one-way ANOVA followed by post-hoc pairwise comparison of all genotypes tested (see left panels of Figs S10, S11). (G) Side and apical views of a 38-day-old *te234* mutant plant grown in long days. Scale bars: 1 cm in D,G; 10 cm in E.

The strong expression of *ECT2* and *ECT3* in early-stage floral primordia led us to examine possible roles of ECT2 and ECT3 in phyllotaxis, i.e. the arrangement of lateral organs on the stem, as that is determined by the sites of primordium initiation. In *Arabidopsis*, the two cotyledons and the first pair of true leaves exhibit an opposite decussate pattern: 180° from one another, and in a 90° twist from the preceding pair. From the third leaf onwards, new organs emerge one at a time forming a spiral with a divergence angle of ∼137.5° (the golden angle; Lüttge and Souza, 2019), albeit with some stochastic variability (Mirabet et al., 2012). To characterize phyllotaxis quantitatively, we measured divergence angles between successive flowers of wild type and two different allele combinations of *ect2/ect3/ect4*. The full circle was divided into 16 intervals of 22.5° (i1-i16), such that 0° falls in i1, the golden angle in i7 and 180° in i9 (Fig. 7A) (Prasad et al., 2011). We assigned each measurement to an interval and calculated their frequencies (f), resulting in the distributions shown in Fig. 7B. Although the wild-type distribution peaks sharply in i7 as expected, *te234* and *Gte234* distributions have an additional prominent peak in i9, indicative of organs diverging by 180° from one another almost as frequently as by 137.5° (f_i7_/f_i9_∼11 in wild type versus f_i7_/f_i9_∼1.5 in *ect2/ect3/ect4*; *P*<0.0001 for both allele combinations, see Materials and Methods). These observations establish that *ect2/ect3/ect4* triple mutants exhibit defective floral phyllotaxis, and therefore imply defects in meristem function, perhaps related to auxin distribution or responsiveness.

Phyllotaxis has not previously been examined in m^6^A writer mutants. Therefore, to assess whether the phyllotaxis defect of *ect2/ect3/ect4* mutants may be connected to m^6^A, we measured divergence angles in *hakai-1* knockout mutants, with m^6^A/A ratios 35% lower than those of wild type (Růžička et al., 2017), and in a more severely m^6^A-deficient transgenic line with low post-embryonic MTA expression in the *mta-1* knockout background (*ABI3pro:MTA/mta-1*) (Bodi et al., 2012). Clear phyllotaxis defects were observed in both cases (Fig. 7C). The defects were more pronounced in *ABI3pro:MTA/mta-1*, with a decrease in f_i7_/f_i9_ almost identical to what we found in *ect2/ect3/ect4* plants, while *hakai-1* mutants had intermediate values [f_i7_/f_i9_∼8 in wild type versus f_i7_/f_i9_∼3 in *hakai-1* (*P*<0.001), and f_i7_/f_i9_∼1.3 in *ABI3pro:MTA/mta-1* (*P*<0.0001), see Materials and Methods]. We also observed additional defects typical of m^6^A deficiency in main stems of *hakai-1*, albeit with low penetrance (see Fig. S8 in the supplementary material). Finally, although we attempted to measure phyllotaxis in *MTA* knockdown plants expressing an artificial microRNA directed against *MTA* (*amiR-MTA*; Shen et al., 2016), the low number of individuals producing stems and their extremely short to non-existing internodes made the quantification impossible. Nevertheless, a clear defect could be visually determined (Fig. 7D). We conclude from these observations that m^6^A is required for normal phyllotaxis, and that ECT2, ECT3 and ECT4 are major effectors of this function.

### Control of flowering time and stem growth are defective in *ect2*/*ect3*/*ect4* mutants

We next examined whether ECT2, ECT3 and ECT4 might influence the transition from vegetative to reproductive meristem (flowering time) and stem growth, as casual observation of *ect2/ect3/ect4* mutants revealed late bolting and shoots shorter than those of wild-type plants at any given time (Fig. 7E; Fig. S9). However, measuring such traits in plants with slow growth altogether is not trivial, as late flowering and shorter stems might be explained by the previous delay in rosette development. To circumvent that problem, we focused on two well-defined points in development: at the time of flowering, we counted number of leaves (NL) and number of days after germination (DAG), and at the time of opening of the 10th flower, we measured the main stem length (SL) and counted the number of days after bolting (DAB) (see Materials and Methods for additional details). The combination of these measurements also allowed us to calculate the rate of leaf production until the floral transition (number of leaves per day, NLD=NL/DAG), and the stem growth per day (SGD=SL/DAB) during the maturation of the first 10 flowers. We measured these parameters in a complete collection of single, double and triple *ect2*, *ect3* and *ect4* mutants, along with transgenic lines expressing wild-type or cage-mutant transgenes of ECT2 and ECT3 in a *te234* background. The results show that *ect2/ect3* and *ect2/ect3/ect4* mutants exhibit defective timing and growth rate during the reproductive phase of development (Fig. 7F). These effects manifest themselves as: (1) early flowering in terms of plant maturity, i.e. with fewer rosette leaves; (2) delayed flowering measured in time, likely as a result of a lower rate of leaf production; (3) reduced stem growth; and (4) slower maturation of flowers, although this latter phenotype only reached formal statistical significance in *te234* mutants. Some single and double mutant combinations other than *ect2/ect3* also showed differences from wild type in varying subsets of these parameters, and, importantly, always with the same tendency as that seen in *ect2/ect3* or *ect2/ect3/ect4* mutants, albeit generally with less significance and/or less pronounced difference (see Figs S10 and S11). Of note, complementation by the wild-type *ECT2/ECT3* genes, but not their m^6^A-binding deficient variants, was clearly observed for the parameters DAG, NLD, SL and SGD (see Figs S10 and S11), establishing that functions of ECT2/ECT3 in control of flowering time and stem growth also require m^6^A-binding activity. Finally, we also sporadically observed defects in the initial direction of the growth of the stem in *te234* mutants (Fig. 7G), resembling the gravitropic defects seen in roots (Fig. 4). In summary, the slow growth of the main inflorescence expands our earlier observations of delayed growth to include not only leaves (Arribas-Hernández et al., 2018) and roots (Fig. 4D), but all vegetative aerial parts and reproductive tissues. Hence, the m^6^A-ECT2/ECT3/ECT4 module is generally required for organogenesis.

### m^6^A-binding capacity of ECT2, ECT3 and ECT4 is required for correct floral patterning

**Fig. 8.**
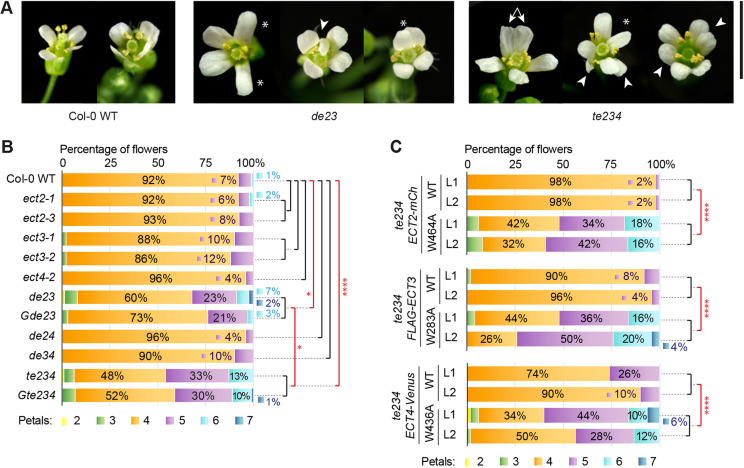
**Defective flower morphogenesis in *ect2/ect3/ect4* mutants.** (A) Representative images of flowers of the indicated genotypes. Asterisks: misplaced and/or mis-shaped petals; arrowheads: supernumerary petals; double arrows: fused petals. Scale bar: 5 mm. (B,C) Number of petals in the first 10 flowers of main inflorescences of 4-10 plants of the indicated genotypes (B) and transgenic lines (C). Percentages are either overlaid or next to their corresponding bars according to graphic convenience, except for flowers with two or three petals, the exact percentages of which are not indicated for simplicity. *P<0.05, ****P<0.0001, proportional odds ordinal regression with post-hoc chi-squared testing. Data corresponding to alleles of the same gene (B), or independent lines expressing the same transgene in the same genetic background (C) were combined, as no significant differences (black brackets) were found between them.

As our RNA *in situ* hybridizations revealed high *ECT2/ECT3* mRNA abundance in all floral organ primordia (sepals, petals, stamens and ovules) at early stages, we investigated whether ECT2, ECT3 and ECT4 are necessary for correct floral patterning. Indeed, preliminary inspections revealed defects in the number, morphology and disposition of petals and stamens in *ect2/ect3* and *ect2/ect3/ect4* mutants (Fig. 8A). In particular, petals were often misplaced from the characteristic cross-disposition in Brassicaceae (Cruciferae), and showed aberrant morphology or inverted orientation (pointing inwards) (Fig. 8A). We chose petals to quantify floral defects, as their size and accessibility allow for a quick assessment of their number. We counted the number of petals in the first 10 or fewer flowers of main inflorescences of combinations of single, double and triple *ect2*, *ect3* and *ect4* mutants. For *ect2*/*ect3*/*ect4*, we could not always include 10 flowers because some plants produced fewer flowers than that. First, we combined data from different alleles of the same genes after verifying the absence of significant differences in petal numbers between them (*P*=0.76, see Materials and Methods). We then tested differences in petal numbers between wild type and each combination of *ect2*, *ect3* and *ect4* mutants. The analysis revealed a significant difference in the number of petals of *ect2/ect3* and *ect2/ect3/ect4* plants compared with wild type, with five- and six-petaled flowers being more frequent in the mutants (Fig. 8B). Additional mutation of *ECT4* significantly exacerbated the defects of the two *ect2/ect3* double mutants (Fig. 8B). Importantly, correct floral patterning requires the m^6^A-binding activity of ECT2, ECT3 and ECT4, because expression of wild type and the cage-mutant transgenes in *te234* yielded highly significant differences (Fig. 8C), whereas no differences were detected in comparisons between the complemented lines and their double mutant equivalents (*de34* for *te234/ECT2-mCherry*, *de24* for *te234/FLAG-ECT3* and *de23* for *te234/ECT4-Venus*, *P*>0.05 in all cases; Fig. 8B,C). Interestingly, we observed a tendency of cage-mutant transgenes to exacerbate the *te234* petal phenotype (Fig. 8B,C), significant for *te234/FLAG-ECT3^W283A^* lines (*P*=0.014). Such dominant-negative effects may arise by competition for binding to other effectors of the m^6^A pathway through interactions via their intrinsically disordered regions (IDRs).

### ECT2 and ECT3 play a role in the determination of fruit shape and size that is dependent on their m^6^A-binding capacity

**Fig. 9.**
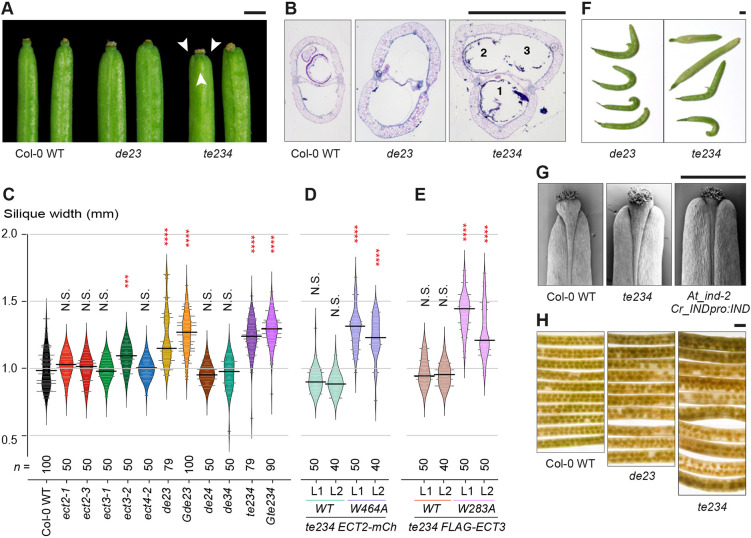
**Fruits of *ect2/ect3/ect4* mutants exhibit aberrant morphology.** (A,B) Characterization of mature siliques of wild-type, *de23* and *te234* plants grown in long days. (A) Photographs of the distal parts of the siliques. Arrowheads indicate carpels in a tricarpelar *te234* fruit; (B) histological cross-sections of the medial part of the siliques. Carpels 2 and 3 are partially fused, i.e. not separated by a septum. (C-E) Violin plots (horizontal black lines represent the medians) showing the distribution of the widths of mature siliques of the indicated mutant alleles or allele combinations (C) and transgenic lines (D,E). Number of measured siliques (*n*) is indicated. ***P<0.001, ****P<0.0001, Kruskal-Wallis rank sum test followed by a Wilcoxon signed rank pairwise comparison between the marked genotype and Col-0 wild type (C) or the marked transgenic line and the matching mutant background, i.e., *de34* in D and *de24* in E. (F) Siliques with aberrant morphology. (G) SEM images of the distal part of siliques. (H) Seeds inside siliques after clearing the carpels. Scale bars: 1 mm.

We finished our analysis by examining fruits of *ect2/ect3* and *ect2/ect3/ect4* mutants. The siliques of *de23* and *te234* mutants were wider than in wild type and, particularly in the triple mutant, they sometimes contained three carpels that could be either completely separated or partially fused (Fig. 9A,B). This increase in fruit width was statistically significant (Fig. 9C) and was mainly due to a lateral expansion in the surface of the carpels (Fig. 9B), reminiscent of the wider laminas observed in juvenile leaves (Fig. 2B). No consistent abnormalities in fruit width could be observed for other *ect* mutants [e.g. the effect seen in *ect3-2* was not corroborated by the other *ect3* allele (*ect3-1*)]. As with all other phenotypes tested, aberrant silique width in *te234* was rescued by expression of wild type, but not m^6^A-binding deficient *ECT2/ECT3* transgenes (Fig. 9D,E).

Although we did not find significant differences in fruit length that were consistent among different combinations of *ect2*, *ect3* and *ect4* mutants compared with wild type, we observed a higher frequency of aberrant fruits exhibiting lengths smaller than 10 mm (see Fig. S12 in the supplementary material), and/or distorted shapes (Fig. 9F) in *ect2/ect3* and *ect2/ect3/ect4* mutants. Furthermore, close inspection of the distal part of *te234* fruits by scanning electron microscopy (Fig. 9G) revealed that the valve tips often extended their apical growth, a characteristic that is pronounced in close relatives of *Arabidopsis* with heart-shaped siliques, such as members of the *Capsella* genus. Such overgrowth has been associated with sequence variation in the regulatory domains of the fruit-tissue identity gene *INDEHISCENT* (*IND*) in *Capsella* species (Dong et al., 2019). Indeed, our analysis shows that the apical overgrowth of siliques from *te234* mutants is reminiscent, albeit milder, of siliques from *Arabidopsis ind-2* mutants expressing *Capsella rubella IND* (Dong et al., 2019) (Fig. 9G).

Finally, we examined the disposition of seeds inside the siliques of *de23* and *te234* mutants by simple inspection of cleared tissue. This analysis revealed that, in both mutants, seeds are placed within the siliques in a more irregular pattern than in wild type (Fig. 9H). In particular, both mutants showed increased occurrence of missing seeds (Fig. 9H), indicative of either defective ovules, failed fertilization or aborted seeds.

## DISCUSSION

### A recurrent role of m^6^A-ECT2/ECT3/ECT4 in plant organogenesis: an accelerator of primed stem cell proliferation?

The main conclusion of the present work is that the m^6^A-ECT2/ECT3/ECT4 module has a ubiquitous role in plant organogenesis: *ect2/ect3* and/or *ect2/ect3/ect4* mutants exhibit specific defects in the architecture of leaves, stems, flowers, fruits and roots, which are formed with a delay. Importantly, these defects can be rescued by wild type, but not by m^6^A-binding deficient mutants, providing a strong argument that defective reading of at least part of the m^6^A program by ECT2, ECT3 and ECT4 causes aberrant development. Organogenesis involves both establishment of a population of primed stem cells deriving from pluripotent meristems, and coordinated cell division, differentiation and expansion in these newly established organ primordia. It is, therefore, of crucial importance for the understanding of the biological relevance of the m^6^A-ECT2/ECT3/ECT4 program to define which of these processes are under its control. Here, we propose that promoting cell division in organ primordia is the key function of this subclade of m^6^A readers for three reasons. First, it is consistent with our expression analyses of ECT2, ECT3 and ECT4 that show highest expression in the rapidly dividing cells of all organ primordia and division zones examined. Second, leaf primordia form roughly at the same time in wild type and *ect2/ect3/ect4* mutants, but the cell division rate in young leaf primordia is reduced compared with wild type. Third, vascular stem cells are less numerous in hypocotyls of *ect2/ect3/ect4* seedlings than in wild type, further proving proliferation defects. We note that stimulation of cellular proliferation by m^6^A-YTHDF2 has also been observed in early zebrafish embryos (Zhao et al., 2017) and in mammalian cell culture (Fei et al., 2020). Nonetheless, biologically relevant m^6^A-YTHDF function in animals often involve developmental transitions in differentiation trajectories, and is thought to rely on YTHDF-mediated stimulation of decay of methylated mRNAs encoding key regulatory factors (Ivanova et al., 2017; Li et al., 2018; Zhang et al., 2017; Zhao et al., 2017). Whether the stimulated cell proliferation by m^6^A-YTHDF axes in early vertebrate embryos and in plant organ primordia also reflects similarities at the level of molecular function must await identification of mRNA targets of proven biological relevance in both systems.

### m^6^A-ECT2/ECT3/ECT4: only one of several m^6^A-dependent regulatory axes in development

Mutants with reduced m^6^A levels (Bodi et al., 2012; Růžička et al., 2017; Shen et al., 2016) show striking phenotypic similarities with *ect2/ect3/ect4* mutants. These include delayed emergence of juvenile leaves with deltoid shape and serrated edges (see Arribas-Hernández and Brodersen, 2020 for a direct comparison), slow root and stem growth, root agravitropism, aberrant flower morphogenesis, increased trichome branching and, as shown here, defective phyllotaxis. Additionally, delayed floral transition has been described in mutants of the m^6^A-demethylase ALKBH10B (Duan et al., 2017) and, although the vascular defects described by Růžička et al. (2017) have not been studied in *ect2/ect3/ect4* mutants yet, the defects in vascular stem cell proliferation (Fig. 1C,D) may be indicative of another similarity. These observations highlight the importance ECT2, ECT3 and ECT4 as effectors of the m^6^A pathway in plants. Nevertheless, *mta*, *mtb*, *fip37* and *vir* knockout embryos arrest at the globular stage (Růžička et al., 2017; Vespa et al., 2004; Zhong et al., 2008), and severe post-embryonic m^6^A depletion causes overproliferation of the SAM and strongly delayed initiation of leaf primordia (Shen et al., 2016), phenotypes not observed in *ect2/ect3/ect4* mutants. The most obvious explanation for these differences is the involvement of the remaining YTH-domain-encoding genes. In that regard, it is interesting that cells in the root QC and the organizing center in the IM express *MTA* but not *ECT2*, *ECT3* and *ECT4*, and mRNA-Seq data from sorted root QC cell populations reveals expression of ECT genes other than *ECT2*, *ECT3* and *ECT4* (Brady et al., 2007). Thus, methylated mRNAs in these cells may be regulated by alternative m^6^A readers. This could account, for example, for the above-mentioned differences in SAM size and initiation of leaf primordia. Furthermore, the only phenotypes described for plants with mild m^6^A deficiency that disagree with those of *ect2/ect3/ect4* mutants are bushy rosettes with small and supernumerary leaves, and severe loss of apical dominance as described by Fray and colleagues (Bodi et al., 2012; Růžička et al., 2017), perhaps related to the multiple SAMs reported by Yu's group (Shen et al., 2016). Interestingly, we have seen such phenotypes among a few primary transformants expressing *ECT2/ECT3* transgenes, raising the possibility that the function of other ECTs may be knocked down in these plants, either by competition due to transgene misexpression in the OC, or perhaps due to co-suppression (Napoli et al., 1990) via siRNAs targeting the highly similar mRNA regions encoding YTH domains. In summary, we propose that *ECT2*, *ECT3* and *ECT4* are the main mediators of m^6^A-stimulated proliferation of primed stem cells in organ primordia, but that distinct m^6^A-ECT axes control behavior of organizing centers and pluripotent stem cells in meristems.

### Redundant and specific functions of ECT2 and ECT3

The phenotypic analyses in this and our previous work (Arribas-Hernández et al., 2018) show that defects in leaf, root and stem growth, floral patterning, and silique morphology arise only upon simultaneous knockout of *ECT2* and *ECT3*. Taken together with their overlapping expression patterns, we consider this to be evidence that ECT2 and ECT3 act redundantly to stimulate growth and proliferation in organ primordia. Although hints that ECT2 and ECT3 may not always be fully redundant have also come from the observation that both single knockouts cause mild trichome branching defects (Arribas-Hernández et al., 2018), this cannot be considered proof of non-redundant molecular function, as lower dosage may cause weak phenotypes in either single mutant and a stronger phenotype in the double mutant, as observed (Arribas-Hernández et al., 2018). Here, we provide a clear example in which ECT2 and ECT3 have different, even opposite, rather than redundant, functions: ECT2 promotes leftwards root slanting while the opposite is true of ECT3. Given the exquisite dose dependence on ECT2 of root slanting, root directionality in *Arabidopsis* may be an ideal system for deciphering the *in vivo* relevance of competition between YTHDF proteins, a fundamental issue that also remains controversial in animals (Zaccara et al., 2020). We note in this regard that the different functions of ECT2 and ECT3 may relate to microtubule assembly, as mutations in tubulin subunits as well as microtubule-associated proteins (MAPs) produce either right or left slanting, depending on how the mutation affects the arrangement of protofilaments in cortical microtubules (Furutani et al., 2000; Ishida et al., 2007; Marinelli et al., 1997; Rutherford and Masson, 1996; Thitamadee et al., 2002) (see Smyth, 2016 for a review). Interestingly, mRNAs encoding 14 out of the 15 *Arabidopsis* α and β-tubulin subunits, as well as MAPs known to affect slanting such as SPIRAL1 (Furutani et al., 2000; Nakajima et al., 2004; Sedbrook et al., 2004), carry m^6^A (Parker et al., 2020). Future studies comparing ECT2 and ECT3 target sets might clarify the origin of the distinct root phenotypes of the single mutants.

We also note that the existence of specialized functions of ECT2 and ECT3 is consistent with their sequence and pattern of evolutionary conservation. Although ECT2 and ECT3 belong to the same subclade of YTHDF proteins (Scutenaire et al., 2018), their IDRs that might exert the effector functions (Du et al., 2016; Park et al., 2019; Ries et al., 2019; Wang et al., 2014; Zhang et al., 2019) have different lengths and compositions. Importantly, most dicot genomes encode orthologs of both ECT2 and ECT3 (Scutenaire et al., 2018), supporting the model of at least some distinct functions of biological importance.

### Compensation of reduced proliferation rates may contribute to *ect2*/*ect3*/*ect4* phenotypes

It is a puzzling observation that leaves of *ect2/ect3/ect4* mutants grow larger than wild type despite a reduced rate of cell division at early stages. Cells recruited into leaf primordia first proliferate in coordination with cytoplasmic growth, keeping their size constant, and subsequently enlarge their volumes through cycles of endoreduplication. Although the final size of the leaf is crucially influenced by the duration and efficiency of the proliferation phase (Czesnick and Lenhard, 2015), lateral organs can reach normal dimensions despite impaired cell division thanks to a mechanism called compensation (Foard and Haber, 1961), in which abnormally enhanced cell expansion is triggered by defective cell proliferation in leaf primordia (Tsukaya, 2002). Accordingly, compensation is seen in, for example, mutants with lesions in genes encoding core cell cycle regulators, positive regulators of cell proliferation and ribosomal proteins (Horiguchi and Tsukaya, 2011). Interestingly, mRNAs encoded by many of these genes present m^6^A marks in *Arabidopsis* seedlings (Parker et al., 2020), e.g. *AE7*, *ER*, *FUGU5/AVP1*, *PFL2*, *RPS21B* or *RPS28B*. Thus, it is possible that mis-regulation of these factors causes reduced cell proliferation and compensation in *ect2/ect3/ect4* mutants. Indeed, such an effect would be in line with our observations of occasional larger cells in the concavities of *te234* mutant leaves. Compensation might also explain the expanded surface of the fruit valves and the increased trichome branching of *ect2/ect3* and m^6^A-writer mutants (Arribas-Hernández et al., 2018; Bodi et al., 2012; Scutenaire et al., 2018; Vespa et al., 2004; Wei et al., 2018) if the compensatory cell enlargement is due to overstimulated endoreduplication (Horiguchi and Tsukaya, 2011). Nevertheless, mutants exhibiting compensation typically produce leaves that barely reach wild-type size and do not grow bigger, as in the case of *ect2/ect3/ect4*. An explanation for their larger final size could involve mis-regulation of additional targets that would cause a more extreme cell expansion and/or extension of the proliferation phase. Interestingly, knockdown of *MTA* also results in delayed leaves that resemble those of *ect2/ect3/ect4* mutants, although in this case their size remains small (Arribas-Hernández and Brodersen, 2020). It is possible, however, that a more profound defect in cell proliferation in these mutants may not be fully counteracted by compensation.

### m^6^A-ECT2/ECT3/ECT4 and auxin

As a final note, we wish to point out that the phenotypes of plants defective in the m^6^A pathway described here and previously (Arribas-Hernández et al., 2018; Bodi et al., 2012; Růžička et al., 2017) are very similar to those with impaired auxin function, e.g. defects in gravitropism (Su et al., 2017), phyllotaxis (Bhatia and Heisler, 2018), leaf shape and size (Sluis and Hake, 2015), and floral development (Thomson and Wellmer, 2019). These similarities raise the interesting question of how much of these phenotypes are explained by mis-regulation of key components of the auxin signaling pathway, including auxin biosynthesis factors, transporters and auxin response factors. In this way, our study provides solid guidelines for future molecular and genetic investigations based on identification of direct mRNA targets of the m^6^A-ECT2/ECT3/ECT4 axis.

## MATERIALS AND METHODS

### Oligonucleotide sequences

Sequences of all oligonucleotides used in this study are available in Table S1.

### Growth conditions

Growth conditions are detailed in Arribas-Hernández et al. (2018). Briefly, we sterilized seeds using a 2 min incubation in 70% ethanol followed by 10 min in 1.5% NaOCl and 0.05% Tween-20, two H_2_O washes, and 2-5 days of stratification at 4°C in darkness. Seedlings were germinated and grown on Murashige and Skoog (MS)-agar medium (4.4 g/l MS salt mixture, 10 g/l sucrose and 8 g/l agar; pH 5.7) at 21°C, receiving light intensities of ∼70 μmol m^−2^ s^−1^, and 16 h light/8 h dark supplemental light cycle as default. To characterize root growth, we used 9.5×9.5 cm square MS (1% agar) plates, placed vertically on racks. Short-day conditions were also used as specified in the text, with 8 h light/16 h dark supplemental light cycle. To assess phenotypes of adult plants, 8-day-old seedlings were transferred to soil and maintained in Percival incubators at 21°C-day/17°C-night temperature at a light intensity of ∼100 μmol m^−2^ s^−1^, and the light regime of choice in each case. We used Philips fluorescent tubes TL-D 90 De Luxe 36 W as the light source.

### Plant material

All lines used in this study are in the *Arabidopsis thaliana* Col-0 ecotype*.* The mutant alleles or their combinations were as follows: *ect2-1* (SALK_002225) (Arribas-Hernández et al., 2018; Scutenaire et al., 2018; Wei et al., 2018), *ect2-3* (GK_132F02), *ect3-1* (SALKseq_63401), *ect3-2* (GABIseq_487H12.1), *ect4-2* (GK_241H02), *ect2-1/ect3-1* (*de23*), *ect2-3/ect3-2* (*Gde23*), *ect2-1/ect3-1/ect4-2* (*te234*) (Arribas-Hernández et al., 2018), *wox4-1* (GK_462G01) (Hirakawa et al., 2010), *mta-2* (*emb1706-2*, Line 41510) (McElver et al., 2001), *hakai-1* (GK_217A12) (Růžička et al., 2017) and *ind-2* (Liljegren et al., 2004)*. ect2-3/ect3-2/ect4-2* (*Gte234*) was generated by genetic cross between *Gde23* and *ect4-2* using the same method described by Arribas-Hernández et al. (2018).

The transgenic lines *ABI3pro:MTA/mta-1* (Bodi et al., 2012), *amiR-MTA* (Shen et al., 2016) and *At_ind-2 Cr_INDpro:IND-GFP* (Dong et al., 2019) have been previously described*.* All transgenic lines expressing *ECT2pro:ECT2-mCherry-ECT2ter*, *ECT2pro:ECT2^W464A^-mCherry-ECT2ter*, *ECT3pro:FLAG-ECT3-ECT3ter*, *ECT3pro:FLAG-ECT3^W283A^-ECT3ter*, *ECT3pro:ECT3-Venus-ECT3ter* and *ECT4pro:ECT4-Venus-ECT4ter* are also described (Arribas-Hernández et al., 2018) or generated by floral dip in additional, single, double or triple mutants of *ect2*, *ect3* and *ect4*, using the same plasmids and methods (Arribas-Hernández et al., 2018). Lines expressing *ECT4pro:ECT4^W436A^-Venus-ECT4ter* were produced in the same way with a plasmid derived from pCAMBIA3300-*ECT4pro:ECT4-Venus-ECT4ter* by site-directed mutagenesis, as also described by Arribas-Hernández et al. (2018), with primers LA769-LA770. The mutation was detected by PCR with primers LA771-LA772 followed by restriction digestion with SatI. In general, line selection was first carried out in *ect2/ect3* and *ect2/ect3/ect4* knockout backgrounds and ∼30 T1s were pre-selected based on phenotypic complementation by visual inspection. Subsequently, lines with single insertions, no signs of silencing and comparable levels of the transgenic protein in the T2 generation entered the final selection (Arribas-Hernández et al., 2018). For the transgenic lines in single mutant backgrounds used in this work, we chose lines with expression levels similar to those able to complement the double or triple mutant phenotypes, as complementation of the *ect2*, *ect3* and *ect4* single mutant phenotypes is difficult to screen (trichome branching and root slanting for *ect2* or *ect3*, or none detected so far for *ect4*). Plants co-expressing *ECT2-mCherry* and *DR5:GFP* used for fluorescence microscopy were the F1 progeny of a genetic cross between *DR5:GFP* (Benková et al., 2003) and *ECT2-mCherry*-expressing plants, performed in the same way as the other crosses described here.

### Generation of MTA-FLAG-TFP/ECT2-mCherry transgenic lines

The upstream regulatory elements (1836 nt) followed by the coding sequence of *MTA* (AT4G10760) and its downstream terminator (709 nt) (*MTApro*, *MTA* and *MTAter*, respectively) were amplified from genomic DNA of Col-0 wild-type inflorescences (DNA extraction was performed as described by Arribas-Hernández et al., 2018) by PCR using USER-compatible primers and the KAPA HiFi Hotstart Uracil+ ReadyMix (Roche). The primers were designed to create overhangs compatible with either the PacI USER cassette present in pCAMBIA2300U [pCAMBIA2300 with a double PacI USER cassette inserted between the PstI-XmaI sites at the multiple cloning site (Nour-Eldin et al., 2006)], or with the flanking sequences of a FLAG-TFP double-tag that was amplified from a pCAMBIA3300U-*AGO1pro:FLAG-TFP-AGO1-AGO1ter* plasmid produced in our laboratory (L.A.-H., unpublished work) using the same USER-compatible methodology. To obtain the *MTApro:MTA-FLAG-TFP-MTAter* construct, the fragments were combined and introduced into pCAMBIA2300U by USER cloning (Bitinaite and Nichols, 2009). Kanamycin-resistant colonies were analyzed by restriction digestion and sequencing, and *Arabidopsis* stable transgenic lines were generated by floral dip transformation (Clough and Bent, 1998) of *mta-2/+* plants using *Agrobacterium tumefaciens* GV3101 carrying the pCAMBIA2300U-*MTApro:MTA-FLAG-TFP-MTAter* plasmid. Selection of primary transformants (T1) was carried out on MS-agar plates supplemented with kanamycin (50 mg/l). Additionally, plates contained glufosinate ammonium (Fluka) (7.5 mg/l) to select for the *mta-2* allele. Segregation studies and genotyping of T2 populations (see below) grown on MS-Agar plates supplemented with either kanamycin (50 mg/l) or glufosinate ammonium (7.5 mg/l), and protein blotting of seedling extracts (see below), allowed the isolation of two independent lines with a single T-DNA insertion locus and comparable expression levels of MTA-FLAG-TFP in an *mta-2* homozygous background (see Fig. S2). Both lines complemented the embryo-lethality of the null *mta-2* mutant allele and did not exhibit the obvious developmental defects typical of m^6^A-deficient mutants (Bodi et al., 2012; Růžička et al., 2017; Shen et al., 2016). T2 plants of the two independent lines were crossed to two independent *ect2-1 ECT2-mCherry* lines, and plants in the resulting F1 progeny were used for fluorescence microscopy. Identical patterns of expression were observed for the two different combinations of lines.

### Genotyping

The method used for genotyping all mutant alleles *ECT2*, *ECT3* and *ECT4* is that of Arribas-Hernández et al. (2018). To genotype homozygous *mta*-*2* in lines expressing *MTApro:MTA-FLAG-TFP-MTAter*, we extracted genomic DNA from young leaves in the same way and used primers LA822-LA823 (wild-type band) and LA269-LA823 (T-DNA band) for PCR (see Fig. S2A). Of note, the reverse primer used to detect the wild-type band (LA823) spans the stop codon of *MTA*, annealing to the end of its coding sequence and the beginning of its 3′UTR. Accordingly, the primer LA823 does not anneal to the C-terminally tagged *MTApro:MTA-FLAG-TFP-MTAter*, and therefore it does not detect the wild-type copy of *MTA* contained in the transgene, allowing for unambiguous genotyping of the mutant background in transgenic lines.

### Western blotting

Protein extraction from 10-day-old vertically grown seedlings and western blotting with ECT2 and mCherry antibodies were carried out as previously described (Arribas-Hernández et al., 2018), with the only difference being that ECT2 antisera instead of ECT2 antibodies affinity purified against antigenic peptides were used for ECT2 detection (1:500 dilution). For selection of *MTApro:MTA-FLAG-TFP-MTAter* lines based on MTA-FLAG-TFP protein expression, GFP antisera (Brodersen et al., 2008) were used at 1:30,000 dilution. In all cases, loading is documented by amido black staining of the large subunit of RUBISCO on the same membrane.

### Phenotypic characterization, its representation and statistic analyses

Data shown in the same graphs or photographs within the same panels were obtained, in all cases, from plants grown in individual pots side by side, or from seedlings within the same Petri dishes. Different genotypes were shuffled among the trays or inside the plates to prevent positional bias. We use a logical and coherent color-coding in all graphs to aid the reader: alleles of the same gene(s) are depicted in vivid shades of the same color, and transgenic lines have de-saturated (pastel) colors matching those of the backgrounds resulting after complementation (or non-complementation for cage mutants). All *P*-values resulting from statistic analyses are corrected for multiple comparisons using the Bonferroni method, and their *P*-values are represented as follows *P<0.05; **P<0.01; ***P<0.001; ****P<0.0001. When no significant (N.S.) differences between alleles of the same gene, allele combinations of the same genes, or independent lines expressing the same transgene in the same genetic background were found, their values were combined for a more robust comparison with wild type, other genotypes or other transgenic lines, depending on the case. All statistic analyses were carried out using R programming language and software environment. Additional details for each phenotypic analysis are given below.

### Histology

Seedlings harvested at 2, 3, 4, 5, 6 (meristem and first true leaves) and 10 (hypocotyls) DAG, rosette leaves of adult plants presenting concavities on the surface or their wild-type equivalents (in terms of leaf size), and mature siliques were incubated in Karnovsky's fixative for 2 h and subsequently dehydrated in a graded acetone series (30%, 50%, 70%, 90% and 100%). The plant material was then infiltrated and embedded in Spurr's resin. The samples were sectioned (2 µm) on a SuperNova Reichert-Jung microtome, stained with 0.05% Toluidine Blue-O (pH 4.4), and imaged with a Nikon Eclipse 80i microscope. Statistical analyses were carried out in two different ways, depending on the data. For the size of the SAM, a linear model with genotype as fixed effect was fitted to the measured data. For the number of epidermal cells in the first true leaves and vascular cells in hypocotyls, a generalized linear model with Poisson distribution was fitted to the data, again using genotype as the fixed effect. This was followed by a post-hoc pairwise comparison of genotypes within DAG or cell type. The doubling time of epidermal cells in the first true leaves was calculated from the slopes of the fits to the corresponding models.

### Macroscopic imaging of plant organs

Photographs of seedlings, roots, rosettes, detached leaves, flowers, inflorescences and siliques were acquired with a Leica MZ16 F stereomicroscope mounted with a Sony α6000 camera for specimens smaller than 2 cm, or with a Canon EOS 1100 D when larger. The cameras were used both for illustrations and for measurements taken on the acquired images. All plants and plant organs were photographed fresh without further manipulation except for siliques showing the seeds contained inside, which were cleared in 90% acetone at 4°C until transparent.

### Characterization of leaves

Pictograms of detached leaves were obtained from photographs using the tool ‘Adjust/Threshold’ of the ImageJ software (Schindelin et al., 2012). The area of every leaf (including petiole) was measured with the same software applying ‘Analyze Particles’ to pictograms of the two first pairs of leaves of 8-10 plants for each genotype and time point. All plants were grown in parallel and the same plants were used to quantify the surface of the first and the second pair of leaves, allowing for direct comparison between data displayed on the two different graphs. As the leaves had to be detached to allow quick and accurate quantification of their surfaces, we used a new set of plants for every 2 days-spaced sampling. Thus, negative oscillations in leaf size over time are due to natural variability among plants of the same genotype, rather than to leaf shrinkage. For statistical analyses, a linear model was fitted to the surface area data with days after germination (DAG) and genotype as fixed effects. Variance stabilizing transformation was determined for the response variable (leaf area) using the Box-Cox procedure (Box and Cox, 1964). Post-hoc pairwise comparisons were made between the genotypes for each DAG.

### Characterization of roots

The characterization and quantification of root growth were made on data points acquired by marking the position of the root tips as they grew vertically on the surface of square MS-agar plates every 24 h from 2 to 11 days after germination. For every graph/plot, only plants grown on the same plates were considered. To characterize mutant alleles, three genotypes were compared at a time. Sterile seeds were spotted 4.2 mm apart from each other on a single row containing three groups of two consecutive seeds per genotype [3×(2×3)=18 seeds per plate], so that the position of every genotype on the plate alternated to prevent positional bias. For the same reason, half of the plates in each series were placed facing the other half, as edge effects due to proximity to the walls of the growing chamber could potentially introduce a bias in growth directionality. For each series, 10 plates were sown and grown in close proximity, accounting for a maximum of 60 seedlings per genotype. Seedlings that germinated with a delay, whose roots grew inside the agar or whose roots halted growth at any time were discarded. Complementation lines were characterized in the same way but comparing four lines in every series, with two groups of two consecutive seeds per line [2×(2×4)=16 seeds per plate], in a total of 10-22 plates. After 11 days of growth, the plates were photographed from the back to obtain sharp images of the daily marks overlaid on the roots. We used the image processing package FIJI (Schindelin et al., 2012) to obtain (*x*,*y*) coordinates (in mm) of each mark. The coordinates were re-aligned to make the first mark match the (0,0) origin of coordinates, and plotted on a two-dimensional *x*/*y* space (using Excel) overlaying roots of the same genotype. These computer-generated images are mirrors of the photographs that represent the seedlings as having the camera facing the front side of the open plate (Fig. 4A). The coordinates were also used to calculate growth rate (*G*[mm]/day), length *L*[mm] and growth indices (VGI, HGI, HGI_L_ and HGI_R_). VGI (=*Ly/L*) and HGI (=*Lx/L*) were calculated after 11 days of growth as described by Grabov et al. (2005). To better describe horizontal growth, we calculated partial HGIs based on daily increments of growth (for growth between days m and n, HGI_m-n_=*Gx*_m-n_/*G*_m-n_) from days 2-3 to days 10-11, sorted the nine values of every root into negative (left) and positive (right) categories, and summed the numerical values in each category to obtain HGI_L_ and HGI_R_, respectively. For statistical analyses, we conducted one-way ANOVAs to compare the effect of genotypes on the growth indices (VGI, HGI, HGI_L_ and HGI_R_). For growth rate, a linear mixed effect model was fitted to the data with DAG and genotype as fixed effects and plant as random effect to account for the repeated measures on the same plants. Variance stabilizing transformation was determined for the response variable (growth rate) using the Box-Cox procedure (Box and Cox, 1964). When the ANOVA was significant, a post-hoc pairwise comparison was performed to find pairwise significant differences between genotypes.

### Quantification of divergence angles

To describe phyllotactic patterns, the divergence angle between the insertion points of two successive floral pedicels in mature inflorescences was measured as previously described by Peaucelle et al. (2007) using the 16 sectors defined in Fig. 7A, with 0° in the midpoint of interval 1 and the golden angle (137.5) towards the middle of interval 7 (Prasad et al., 2011). To measure, we used the homemade device shown in Fig. S13, inspired by the device described by Peaucelle et al. (2007). Also following Peaucelle's work, the phyllotactic orientation of each plant was set to the direction giving the smallest average divergence angle. For statistical analysis, we carried out χ^2^ tests of the counts of divergence angles in i7 and in i9 between wild type, *te234* and *Gte234* (Fig. 7B), or between wild-type and *hakai-1*, or wild type and *ABI3p:MTA/mta-1* (Fig. 7C), and calculated *P*-values for the resulting i7/i9 ratios.

### Characterization of flowering time, stem growth and flower morphology

Quantification of DAG plus number of leaves at flowering time and stem length plus days after bolting to produce 10 flowers was carried out manually for 8-20 plants per genotype, accounting for a total of 203 plants in the 20 genotypes assessed (see Figs S10 and S11). A plant was considered to be flowering on the day that the elongating stem was visible, and it was considered to reach the 10-flower day when the 10th flower on the main stem was wide open. For statistical analyses, a one-way ANOVA was conducted to compare the effect of genotypes on the dependent variable (NL, DAG, NLD, SL, DAB and SGD). When the ANOVA was significant, a post-hoc pairwise comparison was performed to find significant differences between genotypes.

Quantification of petal number was carried out manually by counting the number of petals produced by the first 10 flowers on the main shoot of 4-10 plants per genotype, accounting for a total of 1395 flowers from 140 plants (as some *ect* mutant plants produced fewer than 10 flowers) in the 24 genotypes assessed. All plants were grown in parallel in identical and individual pots, allowing for direct comparison between data displayed on different figure panels. For statistical analyses, we applied a proportional odds model for ordinal regression using the ordinal package in the R software system. The response variable (number of petals) was on an ordinal scale from three to seven petals. Of note, we removed the only three flowers with two petals from the total of 1395 flowers in the dataset to fit the model. Proportional odds ordinal regression was carried out with random effect of plant. The random effect is used to account for the plant-to-plant variation in the pattern of petal numbers, as we counted petals of several different flowers on each of the individual plants used in the analysis. We verified that mutants containing different alleles of the same genes produced the same pattern of petal numbers (likelihood ratio=1.9, d.f.=4, *P*=0.76) and, therefore, could be combined for subsequent tests.

### Characterization of siliques

To quantify the length and width of siliques, we collected the first 10 mature fruits from the main inflorescence stems of 5-10 plants, placed them on stickers (carpels to the sides and replum up/down) and photographed them. Using the software Image J, we quantified the width of the siliques as the length of a line drawn across the fruit perpendicular to the carpel surfaces at the point of maximum thickness (that was the middle point in Col-0 wild type, but was often located towards one end in *ect2/ect3* and *ect2/ect3/ect4* mutants). Owing to a higher propensity of mutant fruits to bend, we quantified the length of the fruit as the sum of the lengths of two lines, one drawn from one end of the fruit to the point of maximum bent, and another from that point to the other end. As the measurements of silique width displayed heteroscedasticity, we applied a Kruskal–Wallis rank sum test for differences in silique width among genotypes followed by a Wilcoxon signed rank pairwise comparison tests for differences of silique width between genotypes.

### Fluorescence microscopy

Roots were imaged with a Zeiss LSM700 confocal microscope in all cases except for the micrographs of *ECT2mCherry/MTA-FLAG-TFP* co-expressing roots, which were acquired with a Leica SP5-X. To image IMs we also used a Leica SP5-X confocal microscope, equipped in this case with dipping objectives. The only exceptions were the images of IMs expressing *ECT2-mCherry* in Fig. 5B,C that were taken with a Zeiss LSM780 (also with dipping objectives). mCherry was excited using laser light of 555 nm in Zeiss microscopes, or of 570 nm in Leica SP5-X, and is represented in magenta in all the main figures to aid visualization when combined with green. Venus and GFP were excited with laser light of 488 nm in Zeiss microscopes, and of 510 nm (only Venus) in the Leica platform. TFP was excited using argon laser light of 458 nm (only with the Leica SP5-X microscope). Emitted light was captured by the filter configuration pre-programmed for mCherry, Venus, GFP and TFP on the respective microscope software. Confocal *z*-section stacks were collected at 0.5 µm spacing throughout the depth of the tissue. 3D and orthogonal projections of *z*-section stacks and merged images were obtained using ImageJ (Schindelin et al., 2012).

### Scanning electron microscopy (SEM)

Scanning electron microscopy was carried out as described by Dong et al. (2019). Briefly, mature fruits were fixed in formaldehyde and infiltrated under vacuum. The materials were critical-point dried in CO_2_ and spotter coated with gold. The samples were subsequently examined using a Zeiss Supra 55VP field Scanning Electron Microscope with an acceleration voltage of 3.0 kV.

### *In situ* hybridization

Primary and young secondary inflorescences of Col-0 wild-type, *ect2-1* and *ect3-1* plants were fixed and embedded in Paraplast Plus embedding medium (Sigma), cut at 8 µm and hybridized as described previously (Dreni et al., 2007). The *ECT2* and *ECT3* digoxygenin-labeled antisense RNA probes (see Fig. S5C for a schematic overview of their locations) were generated by *in vitro* transcription according to the instructions provided with the DIG RNA labeling kit (SP6/T7; Roche). The templates for the probes, which target the 3′UTR of *ECT2* or the coding sequence of *ECT3*, were obtained from cDNA of Col-0 wild-type inflorescences (obtained as described by Arribas-Hernandez et al., 2018) amplified by PCR with primers LA724-LA725 (*ECT2*) or LA391-MH35 (*ECT3*), cloned into TOPO TA (Thermo Fisher Scientific) in anti-sense direction from the T7 promoter contained in the plasmid, and re-amplified with primers LA333(M13) and LA724 (for *ECT2*) or LA391 (for *ECT3*). Sections were observed using a Leica DM6000 equipped with differential interface contrast (DIC) optics. Of note, we also designed a probe for *ECT4* but it did not produce signal in sections of inflorescences, perhaps due to low *ECT4* expression levels.

## Supplementary Material

Supplementary information

Reviewer comments
