# Supplementary material for: Recurrent requirement for the m6A-ECT2/ECT3/ECT4 axis in the control of cell proliferation during plant organogenesis
Source: Development. 2020 Jul 24;147(14):dev189134. doi: 10.1242/dev.189134 (PMC7390628; doi:10.1242/dev.189134)
Supplement: Supplementary information [file develop-147-189134-s1.pdf]

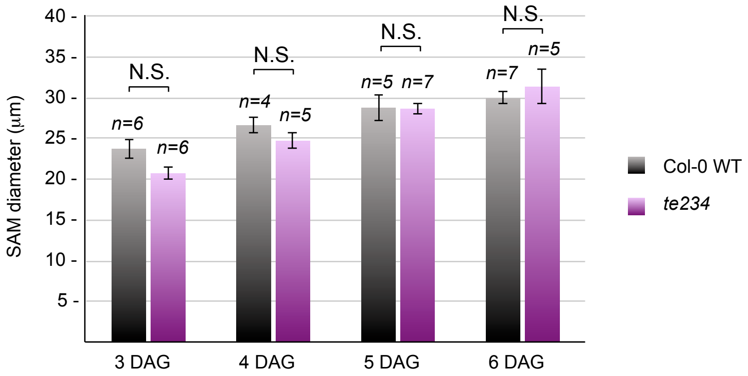

Figure S1. Shoot apical meristems of *ect2/ect3/ect4* mutants do not significantly differ in size with wild type during days 3-6 after germination. Quantification (means±s.e.) of the diameter of the shoot apical meristem (SAM) over time (3-6 days after germination, DAG) in wild type vs *te234* triple mutant seedlings as measured in photopgraphs of longitudinal histological sections. Number of individuals measured (n) are indicated. N.S., not significant ($p > 0.05$, see Methods).

Development • Supplementary information

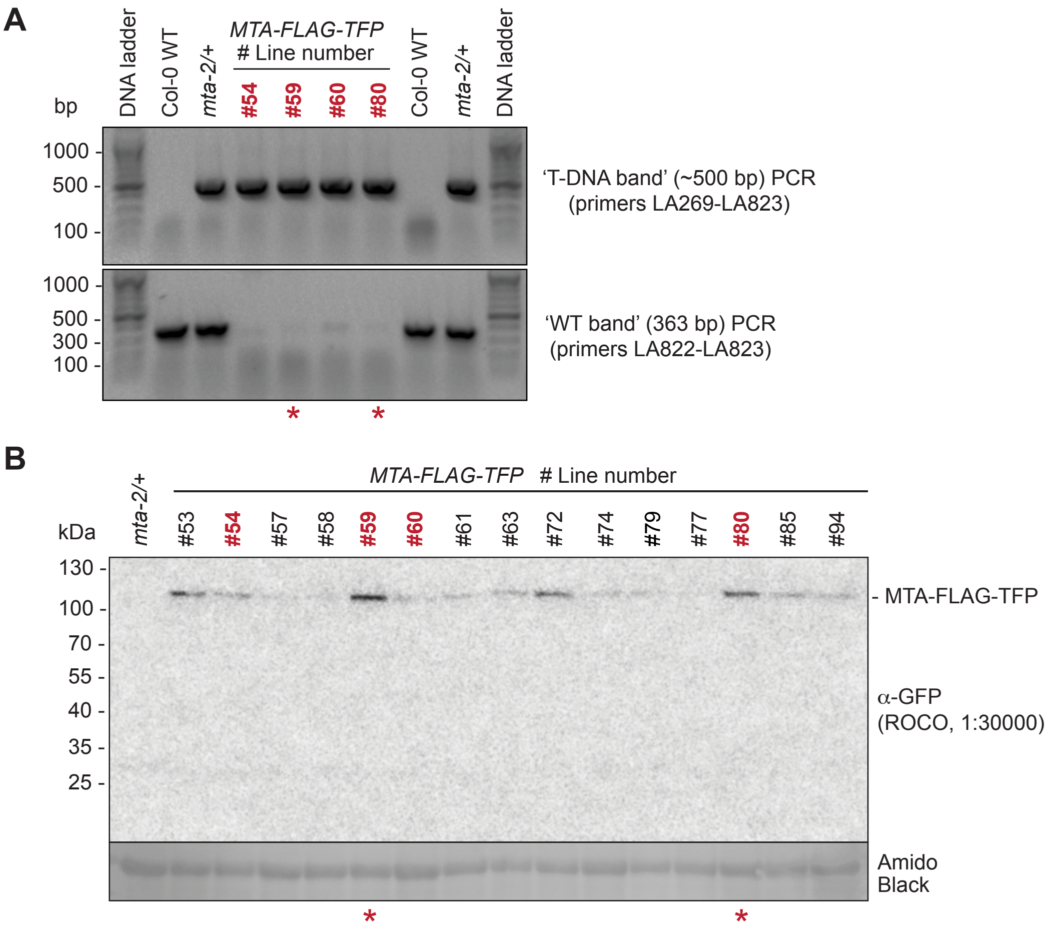

Figure S2. Selection of *mta-2 MTApro:MTA-FLAG-TFP-MTAter* lines. (A) PCR products obtained with the indicated primer sets. The template for PCR was DNA extracted from pools of ~12 seedlings (per line) of the second generation after transformation (T2) of *mta-2/+* plants with *MTApro:MTA-FLAG-TFP-MTAter*. Lines #54, #59, #60 and #80 (highlighted in red) were selected for the analysis based on the T2 segregation on MS-agar plates supplemented with either glufosinate ammonium (selection for *mta-2*) or kanamycin (selection for pCAMBIA2300U *MTApro:MTA-FLAG-TFP-MTAter*). For these lines, 100% of T2 seedlings had resistance to glufosinate ammonium (as expected if *mta-2* is in homozygosity) and to kanamycin (as expected from the progeny of a homozygous *mta-2* T1 plant complemented with the pCAMBIA2300U *MTApro:MTA-FLAG-TFP-MTAter* transgene, as plants without the transgene (kanamycin-sensitive) in the next generation would also be embryo-lethal and not capable of germination). Col-0 WT (non transgenic) plants, and the parental *mta-2/+* line are used as controls for PCR. bp, base pairs.
(B) Protein blot analyses of T2 seedlings described in A. The lines used for the analysis were pre-selected based on visual inspection of TFP fluorescence at the root tips and segregation analyses. Lines other than #54, #59, #60 and #80 showed sensitivity to both glufosinate ammonium and kanamycin in ~1/4 of the population, accounting for *mta-2* heterozygosity and a single insertion of the pCAMBIA2300U *MTApro:MTA-FLAG-TFP-MTAter* transgene. Amido black staining of the membrane is used as loading control.

Lines #59 and #80 (asterisks) were selected to cross to two independent *ect2-1 ECT2-mCherry* lines for fluorescence microscopy studies (Figures 3J-L and 6D,E) as they (i) expressed *MTA-FLAG-TFP* at comparable levels, (ii) were *mta-2* homozygous, and (iii) displayed wild type phenotypes at all developmental stages, proof that the MTA-FLAG-TFP fusion protein expressed in them is functional.

Development • Supplementary information

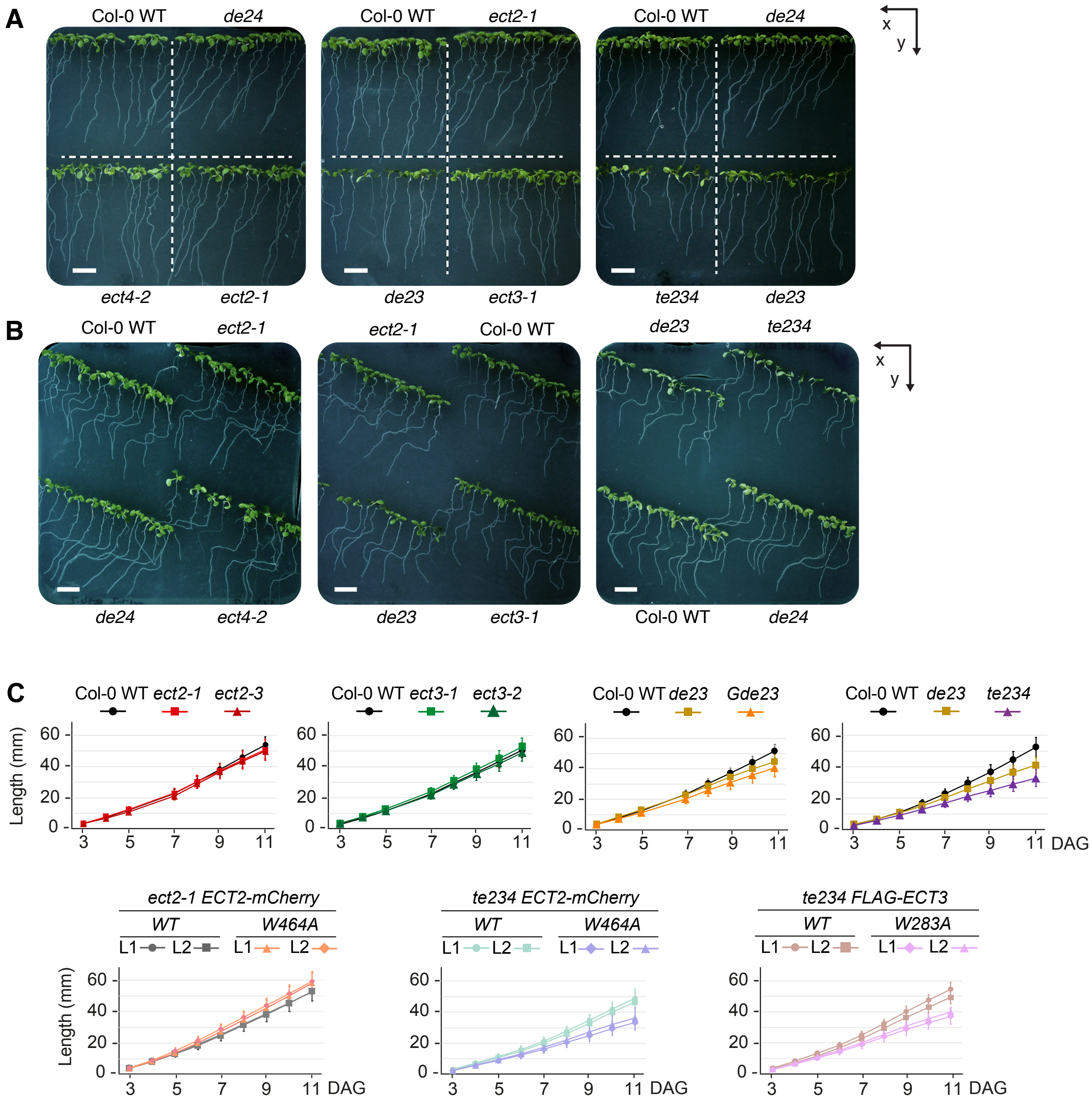

Figure S3. Mutation of *ECT2/3/4* can reduce growth rate and change the gravitropic response of roots (extended data). (A) Photographs of 9-day-old seedlings of the indicated genotypes with their roots grown on the surface of MS-agar inside vertical plates. **(B)** Same as in A, with seedlings grown vertically for 4 days (y axis), turned 90º for 2 days (x axis), and turned again to the original position (y axis) for 3 additional days. **(C)** Root length (L [mm]) (means±s.d.) of the indicated genotypes over a period of 3 to 11 days after germination (DAG). Measurements displayed in the same graphs are taken from seedlings growing with circularly permutated positions on the same plates. Scale bars: 1 cm.

Development • Supplementary information

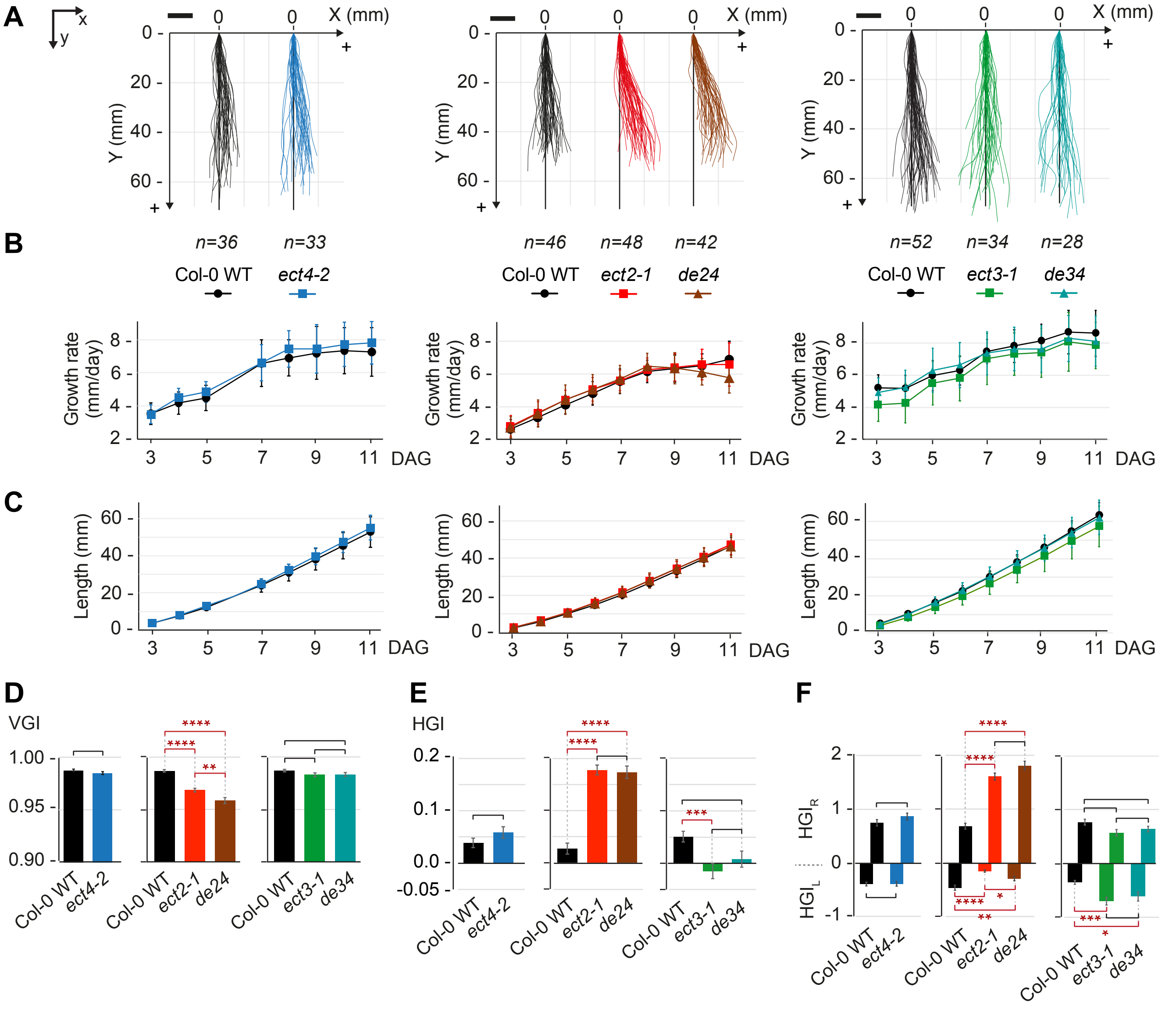

Figure S4. Mutation in *ECT4* alone, or in the individual *ect2-1* and *ect3-1* knockout backgrounds, does not produce substantial changes in root growth. Characterization of roots of the indicated genotypes. **(A)** Representation of the growth pattern on a two-dimentional x/y space as taken with the camera facing the backside of the plate. **(B,C)** Root growth rate in mm/day (means±s.d.) (B) and root length in mm (C) over a period of 3 to 11 days after germination (DAG). **(D,E)** Vertical and Horizontal Growth Indices (VGI and HGI respectively) (means±s.e.) at 11 days after germination, obtained as indicated in Fig. 4B; **(F)** Cumulative left and right Horizontal Growth Indices (HGI_L/HGI_R) (means±s.e.) obtained as described in Fig. 4G. Measurements displayed in the same graphs are taken from seedlings growing with circularly permutated positions on the same plates. Abbreviations, brackets and asterisks are as in Fig. 4.
Scale bars: 10 mm.

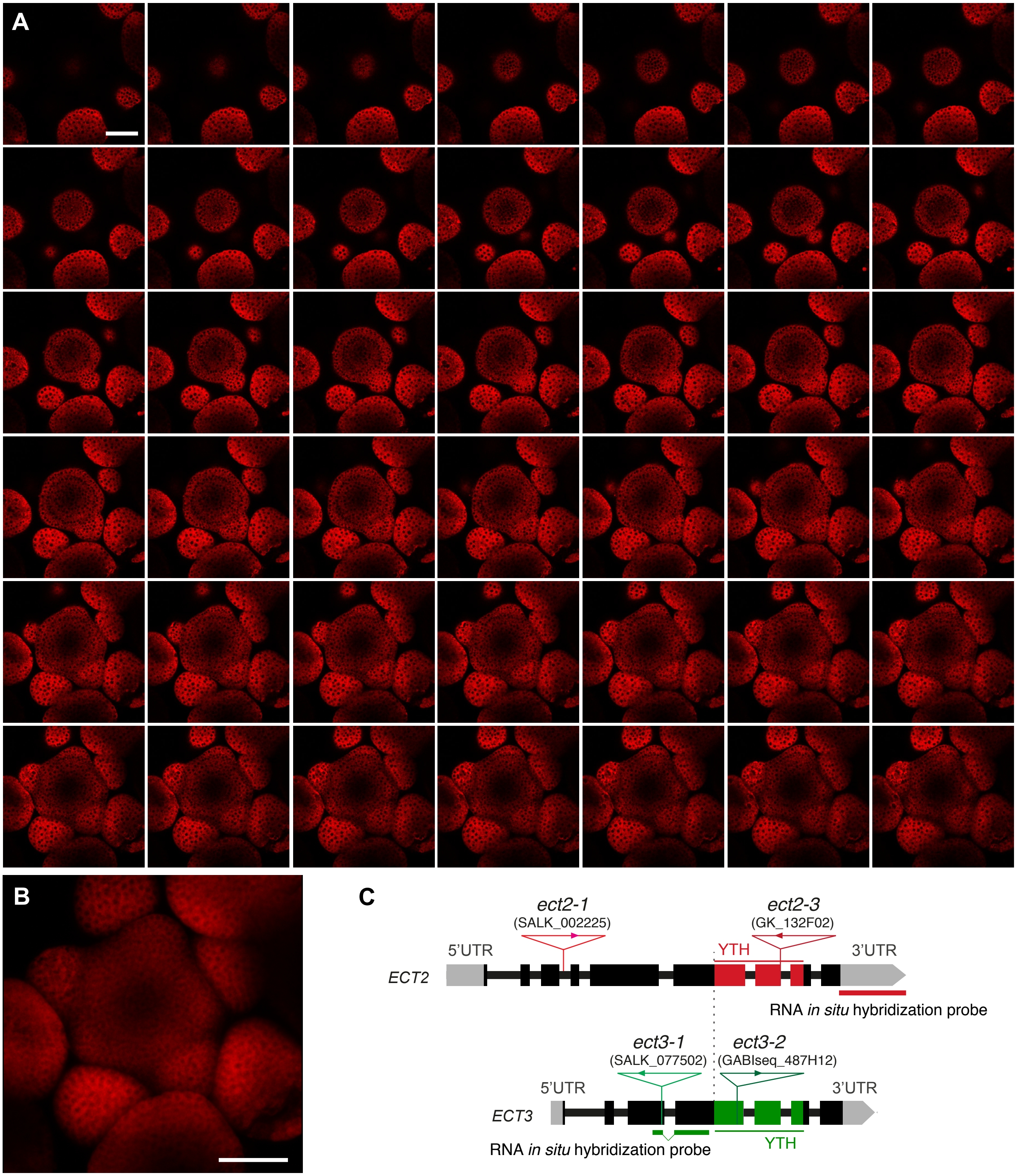

Figure S5. Expression of ECT2 and ECT3 at the inflorescence meristem (extended data). (A) Confocal microscopy images of 42 optical sections (z-stack) covering a total depth of 21 µm of an inflorescence meristem expressing ECT2-mCherry (red colour). The same z-stack was used to illustrate the studies in Fig. 6B,C. **(B)** 3D Projection of 56 optical sections of the z-stack in A (same 42 sections plus 14), covering a total depth of 28 µm of the inflorescence meristem expressing ECT2-mCherry. **(C)** Schematic representation of *ECT2* and *ECT3* mRNAs. The positions of the probes used for RNA *in situ* hybridization (Figures 6, S6 and S7) is indicated, as well as the predicted insertion points of T-DNA in the knockout alleles characterized in this study. Of note, a probe designed for *ECT4* did not provide signal, probably due to low expression levels. Scale bars: 50 µm .

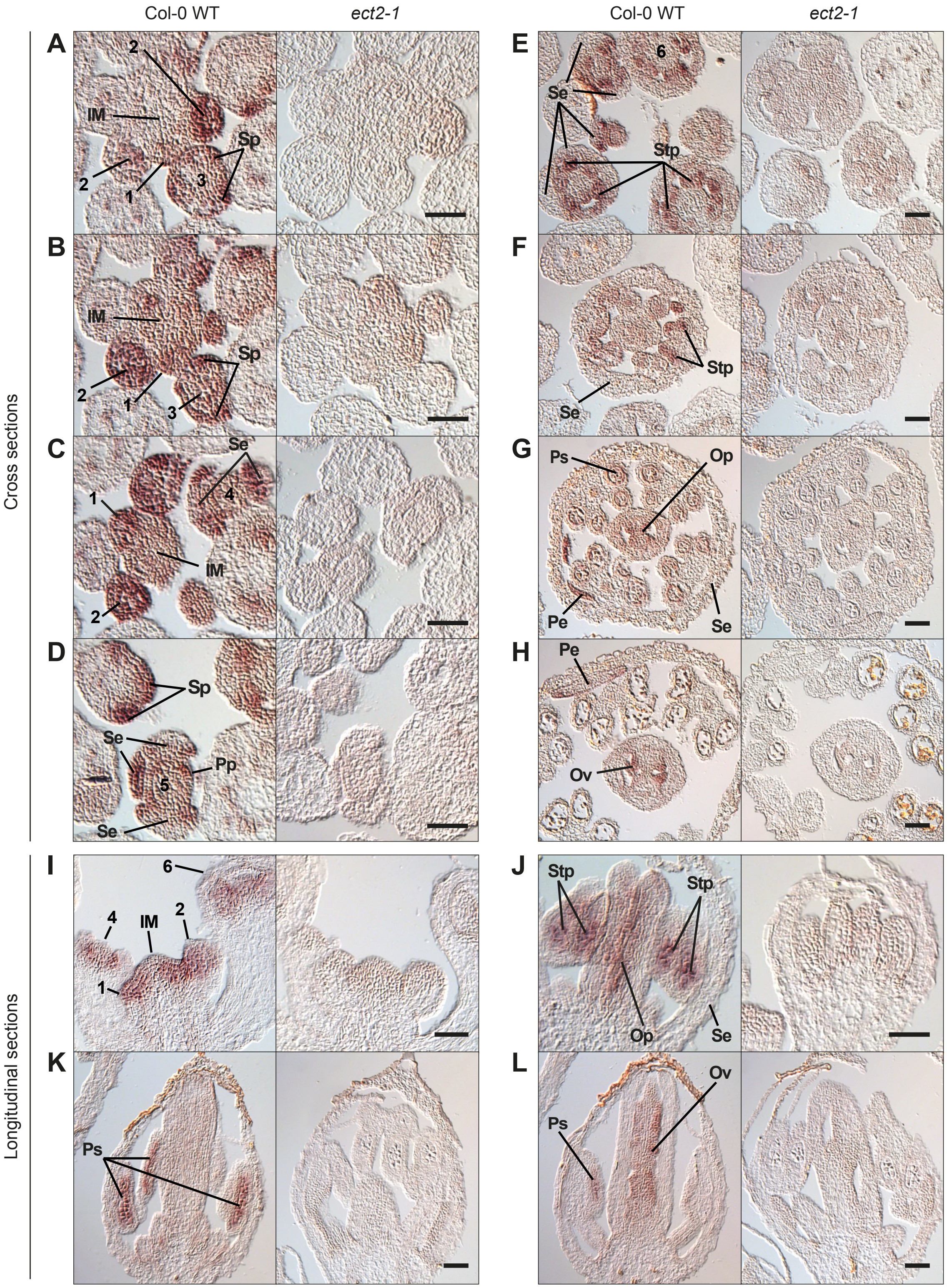

Figure S6. Expression of *ECT2* in inflorescence meristems and floral primordia (extended data). *ECT2* mRNA detected by *in situ* hybridization of tissue sections of Col-0 wild type or *ect2-1* inflorescences as indicated. Hybridization to the antisense probe (Fig. S5C) is revealed by the presence of red colour. The nearly total absence of signal in the *ect2-1* knockout background accounts for the specificity of the probe in these tissues. The two consecutive sections of Col-0 wild type in A and B were used to assemble Fig. 6F. Numbers refer to stages of floral development (Smyth et al., 1990). IM, inflorescence meristem; Sp, sepal primordium; Se, sepal; Pp, petal primordium; Pe, petal; Stp, stamen primordium; Ps, pollen sac; Op, ovule primordium; Ov, ovule. Scale bars: 50 µm.

Development • Supplementary information

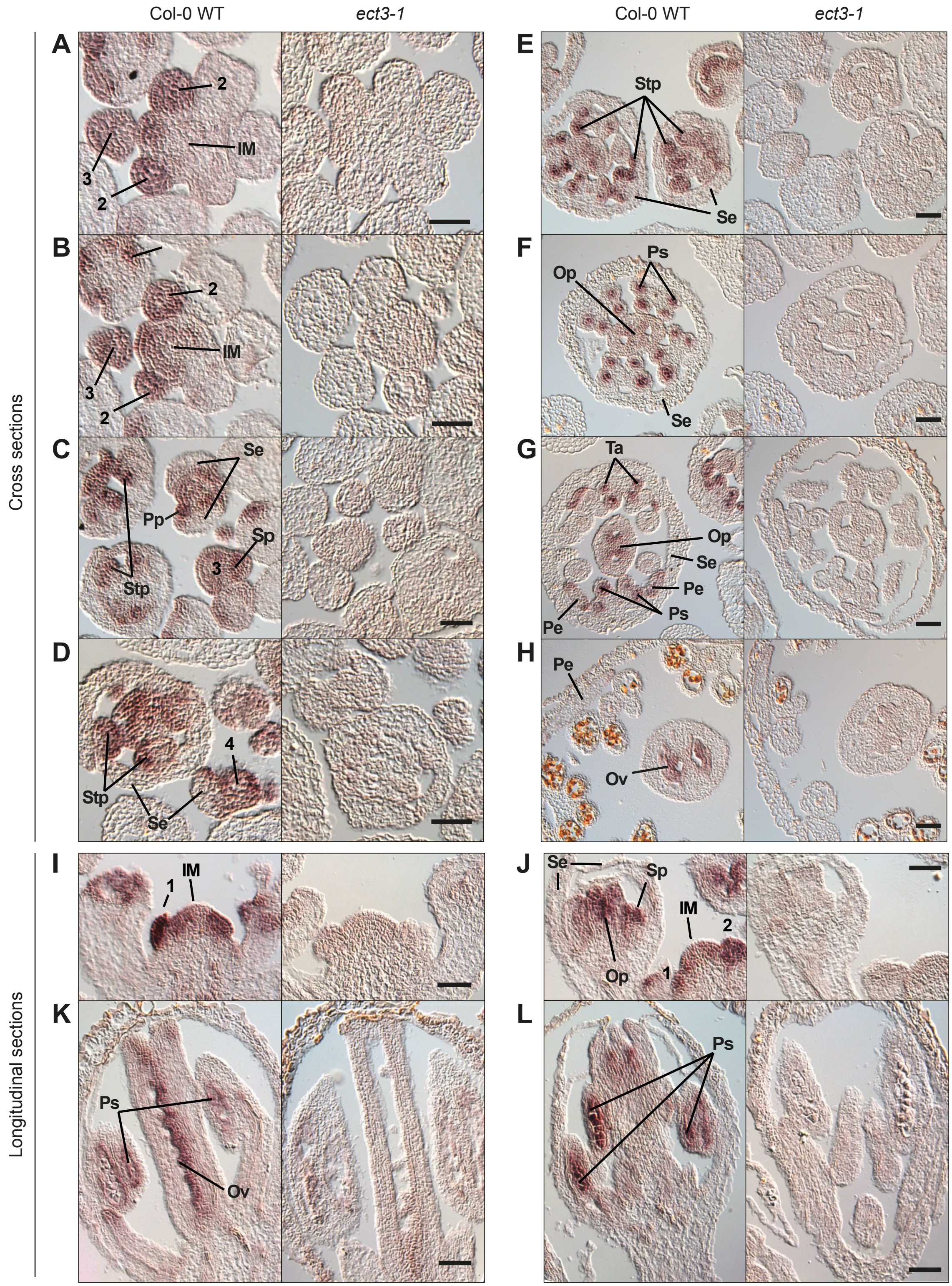

Figure S7. Expression of *ECT3* in inflorescence meristems and floral primordia (extended data). *ECT3* mRNA detected by *in situ* hybridization of tissue sections of Col-0 wild type or *ect3-1* inflorescences as indicated. Hybridization to the antisense probe (Fig. S5C) is revealed by the presence of red colour. The nearly total absence of signal in the *ect3-1* knockout background accounts for the specificity of the probe in these tissues. Numbers refer to stages of floral development (Smyth et al., 1990). IM, inflorescence meristem; Sp, sepal primordium; Se, sepal; Pp, petal primordium; Pe, petal; Stp, stamen primordium; Ps; pollen sac; Op, ovule primordium; Ov, ovule. Scale bars: 50 μm.

Development • Supplementary information

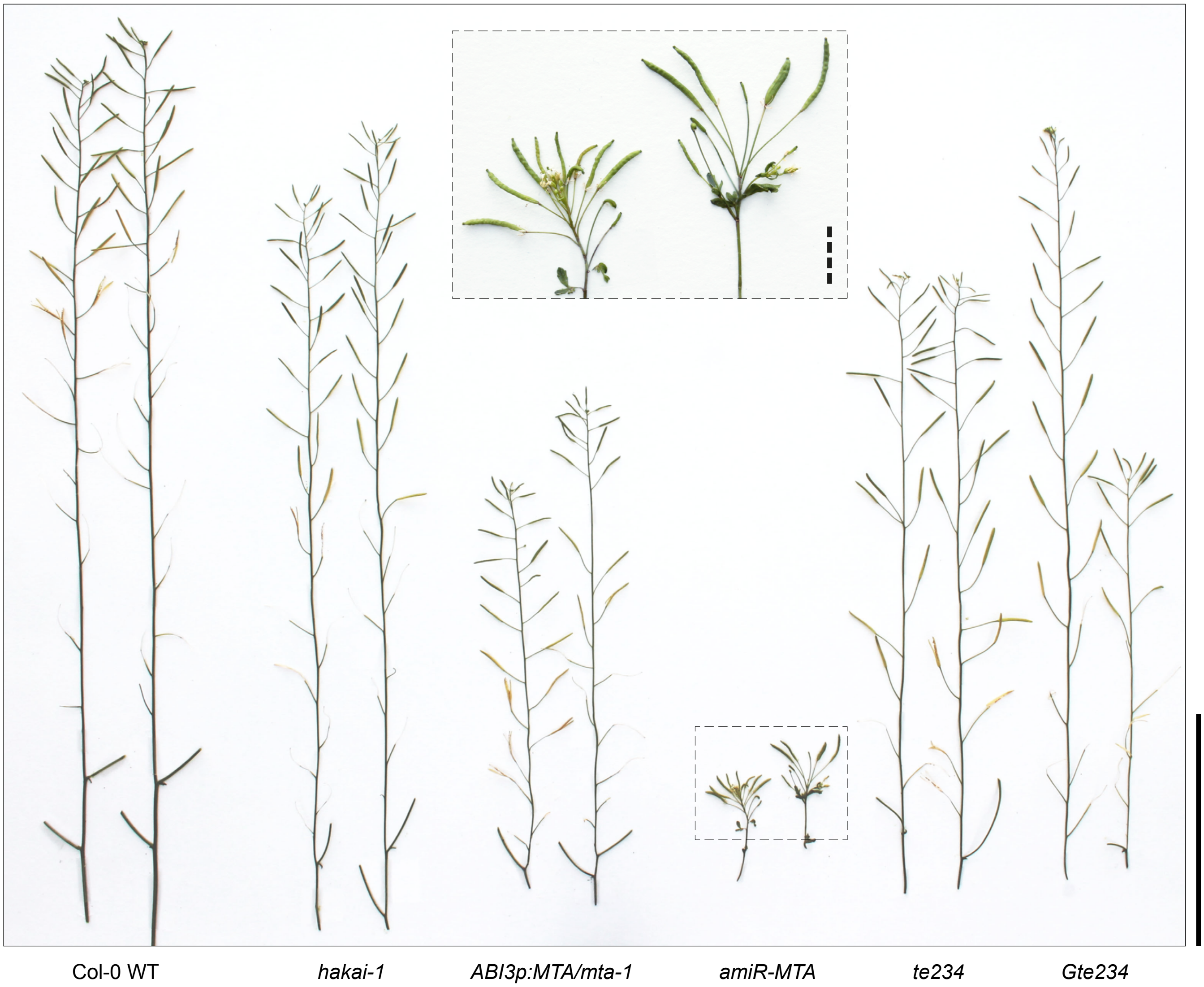

Figure S8. Main stems of the indicated genotypes grown in long days for 55 days. Compared to wild type, *hakai-1* mutants exhibit thinner and shorter main stems bearing more irregularly spaced but generally shorter internodes, and smaller siliques: a milder version of the loss of apical dominance and short fruits displayed by *ABI3p:MTA/mta-1* and *amiR-MTA* (magnified in the dashed box) mutant plants. Main stems of *ect2/ect3/ect4* mutants display variability but generally resemble the defects of m^{6}A writer mutants in terms of loss of apical dominance and aberrant phyllotaxis. Scale bars: whole 10 cm; dashed 1 cm.

Development • Supplementary information

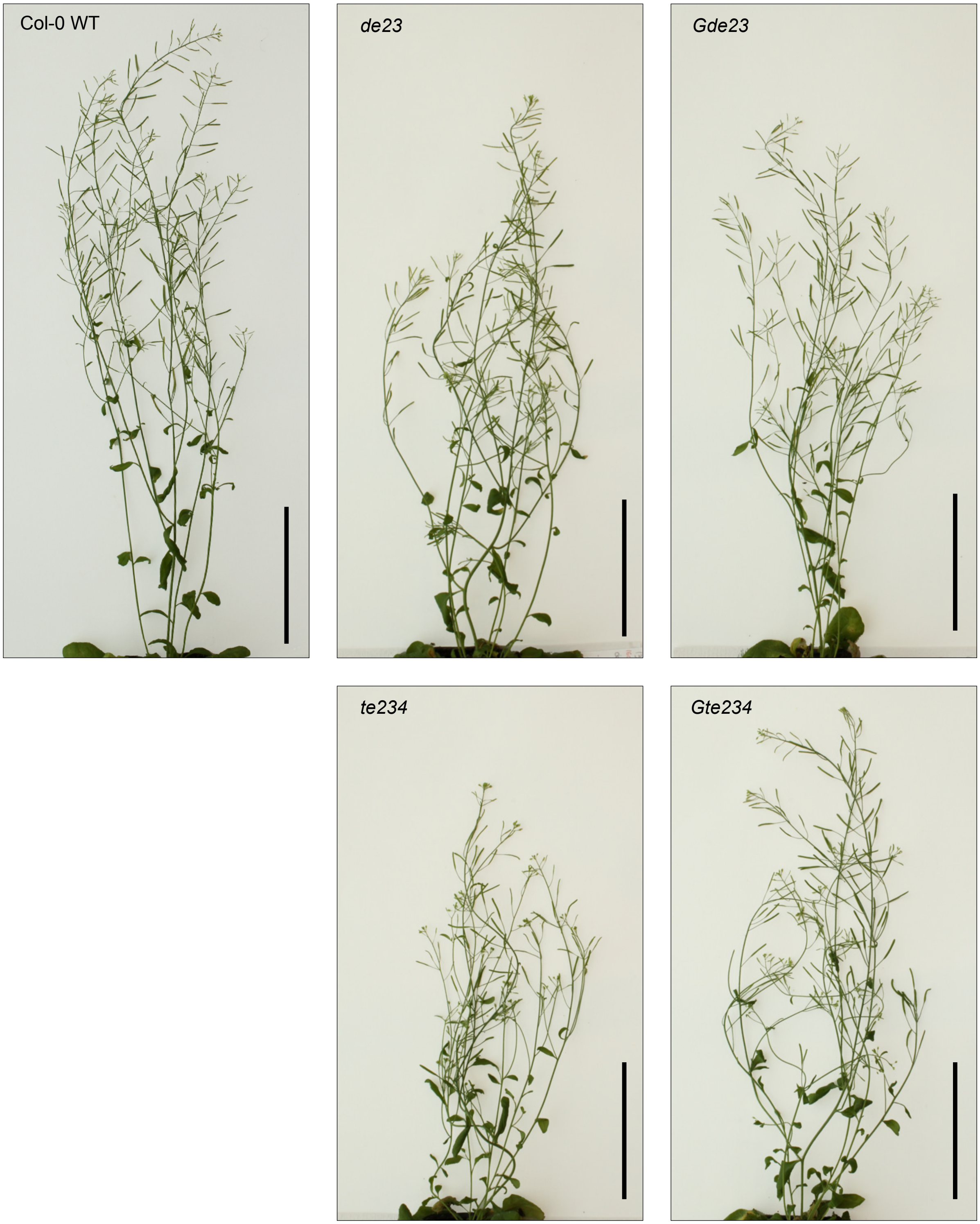

Figure S9. Phenotype of wild type, ect2/ect3 and ect2/ect3/ect4 plants grown in long days for 42 days. Scale bars: 10 cm.

Development • Supplementary information

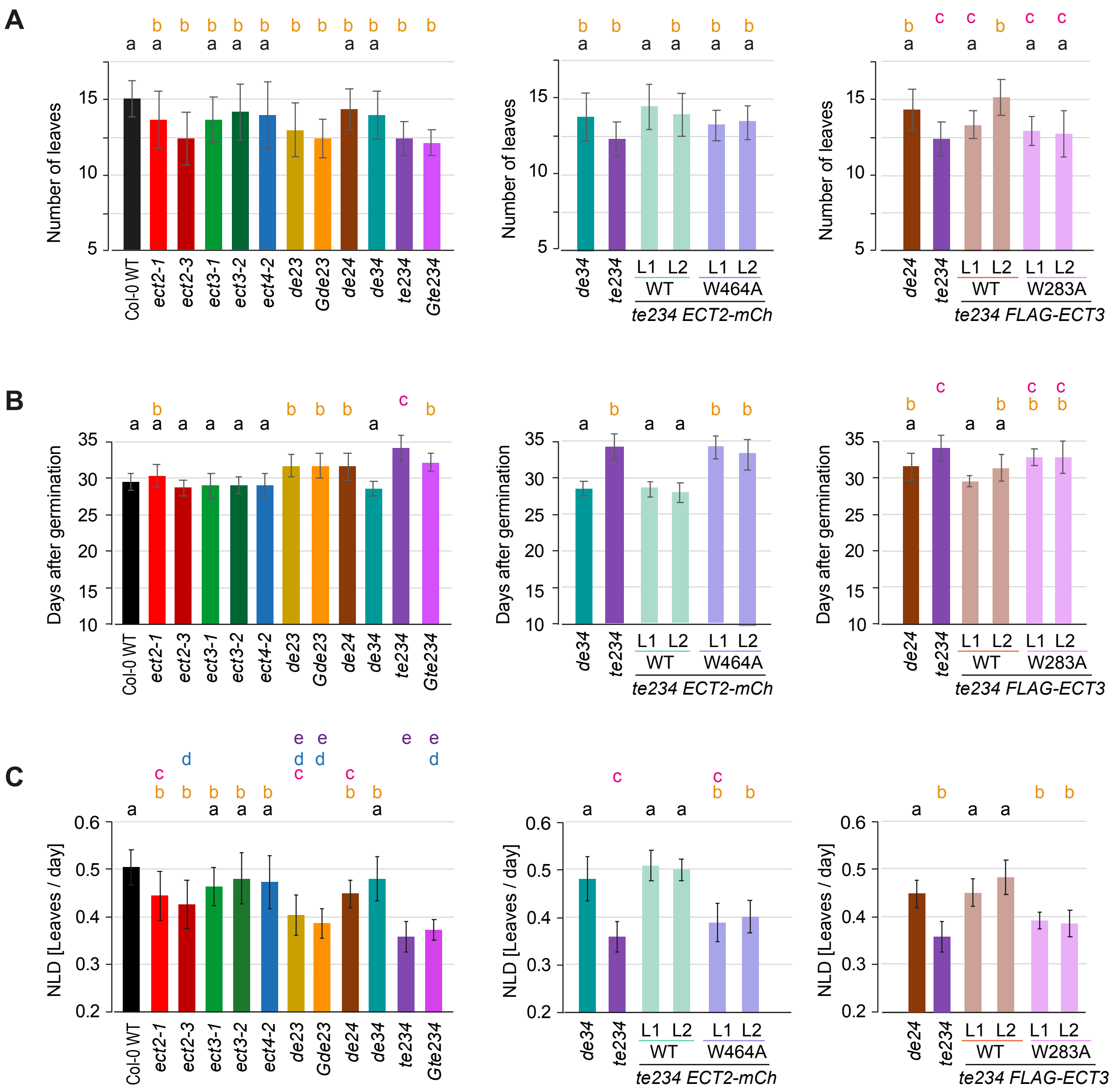

Figure S10. Defective flowering time and leaf growth rate in *ect2/3/4* mutants (extended data). Histograms (means±s.d.) showing the number of leaves **(A)** and days after germination **(B)** at flowering, and the rate of leaf formation during that period of time (number of leaves per day, NLD) **(C)** for plants of the indicated genotypes grown in long days. In each graph, genotypes with the same letter do not differ significantly (Bonferroni corrected *p* value > 0.05 of one-way ANOVA). The *p* values were obtained through post hoc pairwise comparisons of all genotypes displayed in the same graph.

Development • Supplementary information

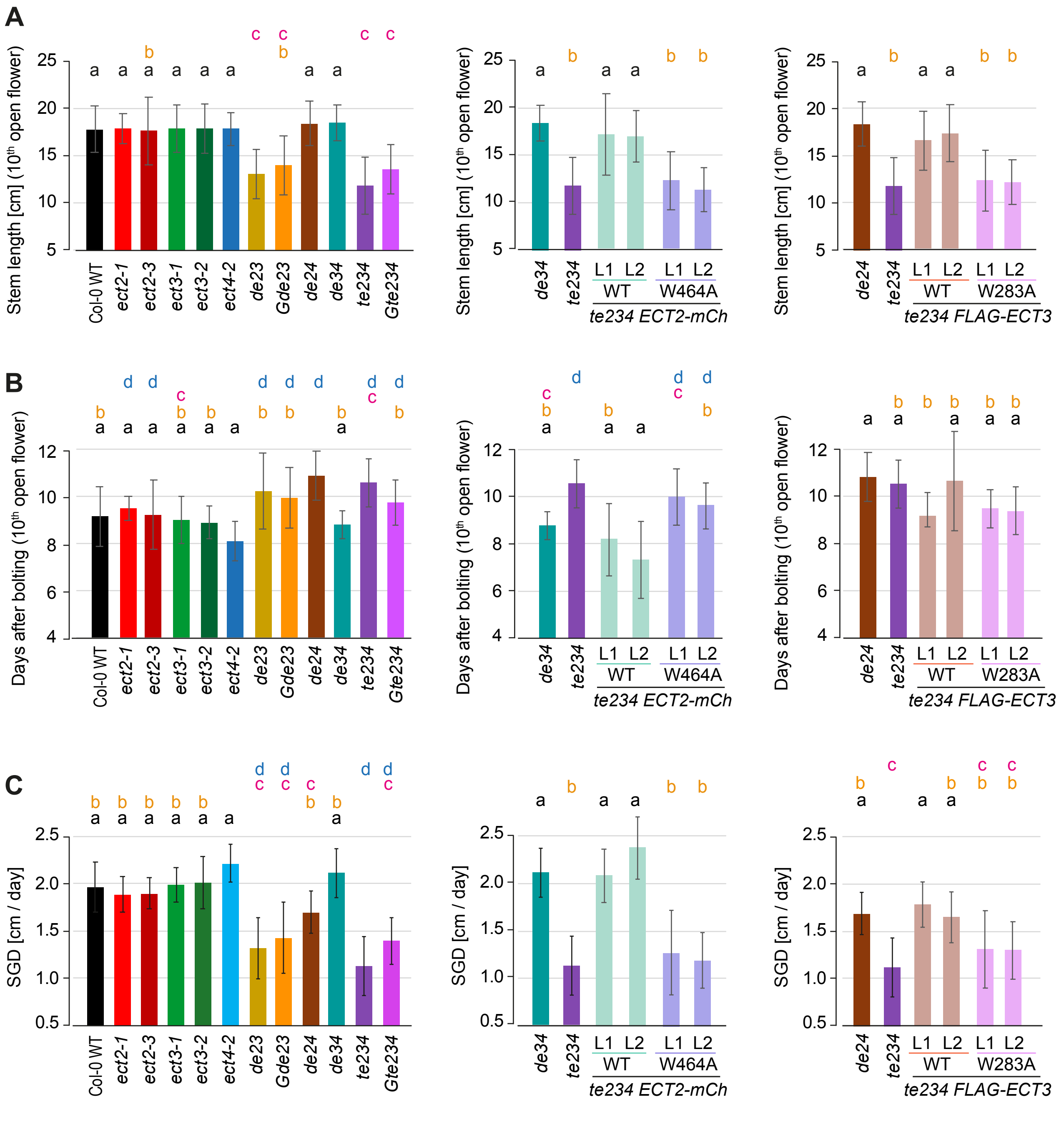

Figure S11. Slow growth of the main inflorescence stem in *ect2/3/4* mutants (extended data). Histograms (means±s.d.) showing the length of the main inflorescence stem [cm] at the time in which the 10th flower opens **(A)**, the time [days] after bolting that it takes **(B)**, and the rate of stem growth during that period (stem growth per day, SGD [cm/day]) **(C)** for plants of the indicated genotypes grown in long days. In each graph, genotypes with the same letter do not differ significantly (Bonferroni corrected p value > 0.05 of one-way ANOVA). The p values were obtained through post hoc pairwise comparisons of all genotypes displayed in the same graph.

Development • Supplementary information

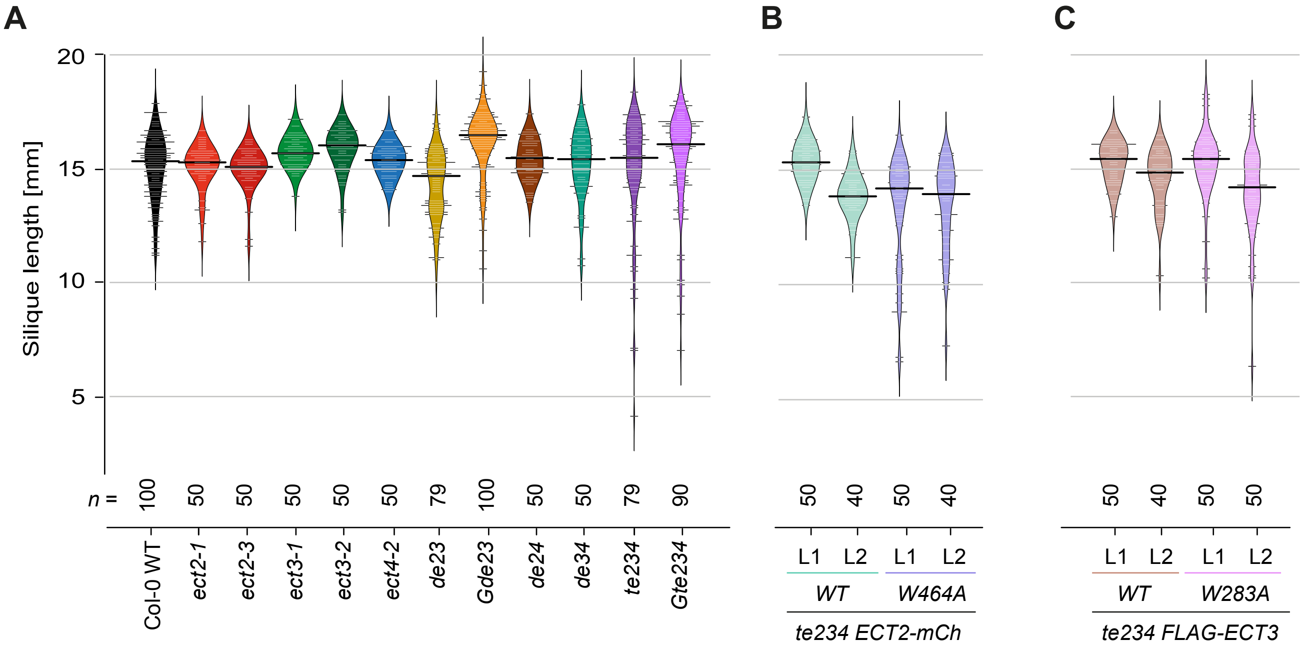

Figure S12. Fruits of *ect2/3/4* mutants exhibit aberrant morphology (extended data). Violin plots ((horizontal black lines represent the medians) (*Adler D. Violin plot. R package version 0.2 (2005); https://CRAN.R-project.org/package=vioplot*) showing the distribution of the length of mature siliques for the indicated mutant alleles or allele combinations **(A)** and transgenic lines **(B,C)**. Notice the higher frequency of aberrant, extraordinarily small siliques (<10 mm), in *ect2/ect3/ect4* mutants with or without m^{6}A-binding deficient *ECT2* (W464A) or *ECT3* (W283A) transgenes.

Development • Supplementary information

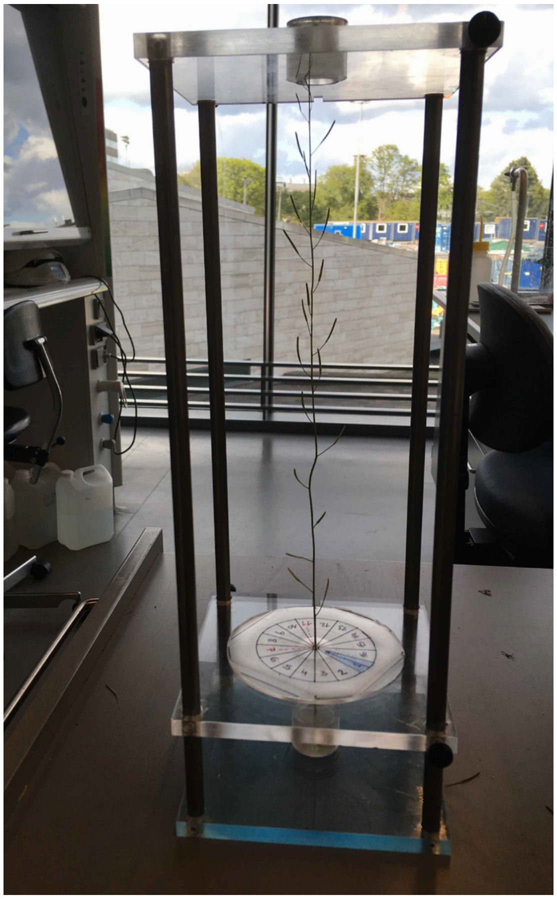

Figure S13. Homemade apparatus to measure phyllotaxis. The design was inspired by the device described by Peaucelle et al. (2007)

Table S1. DNA Oligonucleotides (all sequences are 5' to 3')

Genotyping primers

***mta-2* (*emb1706-2*)**	**WT band (LA822-LA823): 363 bp. T-DNA band (LA269-LA823): ~500 bp**
LA822.*mta-2* .F	TTGTCAACCACCAGATGTATGCAATG
LA823.*mta-2* .R	GGCTTTGTTTTTTTTGGAATTGAACTAAGCTG
LA269.SAIL-LB2	GCTTCCTATTATATCTTCCCAAATTACC

Primers for probes used in RNA in situ hybridization

LA724.ECT2-3‘UTR_probe.F	CTAAGAGGATGGTGTCGCTCAC
LA725.ECT2-3‘UTR_probe.R	TCAACAATACTTATCTCCTTGC
LA391.ECT3-probe.F	TCCCCCAGCATATCAGTACC
MH35.ECT-probe.R	CCTTCAAAGACACAGCCATCAC
LA333.M13.F	CCCAGTCACGACGTTGTAAAACG

Cloning

**Constructs (USER-cloning)**	**Primer pairs (fragments)**
MTApro:MTA-FLAG-TFP-MTAter (in pCAMBIA3300-U)	LA810-LA785 (MTApro), LA786-LA787 (FLAG-TFP), LA788-LA789 (MTAter)

Primers/Oligonucleotides for USER-cloning

LA810.U-MTA_P(1.8Kb).F	GGCTTAAUgatatacactccatcttgttg
LA785.MTA/FLAG.R	AtcAGCUGTGATTGAGTCAATAGCCATTG
LA786.MTA/FLAG.F	AGCTgaUtacaaggatgacgatgacaag
LA787.TFP/MTA_T.R	ACTActtgUacagctcgtccatgc
LA788.TFP/MTA_T.F	AcaagTAGUtcaattccaaaaaaaacaaagccaaatgtctcagc
LA789.MTA_T-U.R	GGTTTAAUCTCGTAAAGACGCTACCAAAGC

Primers for site-directed mutagenesis (QuickChange)

LA769.ECT4_W436A_QC.F	GAGCATCAAATATAATGTAGCGGCTAGCACCCCAAATGG
LA770.ECT4_W436A_QC.R	CCATTTGGGGTGCTAGCCGCTACATTATATTTGATGCTC

Primers for detection of point mutations

LA771.ECT4_W436A_CP(SatI).F	AGAGAAGAGTGCAACAGAGACG
LA772.ECT4_W436A_CP(SatI).R	GCAGCATCAAGCTTCTTGTTTCC
