# Supplementary material for: Recurrent requirement for the m6A-ECT2/ECT3/ECT4 axis in the control of cell proliferation during plant organogenesis
Source: Development. 2020 Jul 24;147(14):dev189134. doi: 10.1242/dev.189134 (PMC7390628; doi:10.1242/dev.189134)
Supplement: Reviewer comments [file develop-147-189134_review_history.pdf]

Recurrent requirement for the m^{6}A-ECT2/ECT3/ECT4 axis in the control of cell proliferation during plant organogenesis

Laura Arribas-Hernández, Sara Simonini, Mathias Henning Hansen, Esther Botterweg Paredes, Simon Bressendorff, Yang Dong, Lars Østergaard and Peter Brodersen
DOI: 10.1242/dev.189134

Editor: Yka Helariutta

Review timeline
Original submission: 6 February 2020
Editorial decision: 5 March 2020
First revision received: 23 May 2020
Accepted: 22 June 2020

Original submission

First decision letter

MS ID#: DEVELOP/2020/189134

MS TITLE: Recurrent requirement for the m^{6}A-ECT2/ECT3/ECT4 axis in the control of cell proliferation during plant organogenesis

AUTHORS: Laura Arribas-Hernández, Sara Simonini, Mathias Henning Hansen, Esther Botterweg, Simon Bressendorff, Yang Dong, Lars Ostergaard, and Peter Brodersen

I have now received all the referees reports on the above manuscript, and have reached a decision. The referees' comments are appended below, or you can access them online: please go to BenchPress and click on the 'Manuscripts with Decisions' queue in the Author Area.

The overall evaluation is positive and we would like to publish a revised manuscript in Development, provided that the referees' comments can be satisfactorily addressed. Please attend to all of the reviewers' comments in your revised manuscript and detail them in your point-by-point response. If you do not agree with any of their criticisms or suggestions explain clearly why this is so.

Reviewer 1

Advance summary and potential significance to field

The post-transcriptionally added m6A modification is required in plants for progression past the globular stage of seed development, and plants with reduced levels of m6A, as seen in hypomorphic mutants of the writer complex have a plethora of developmental defects. The m6A mark is recognised by proteins containing a YTH domain, and compared to mammals, plants contain an expanded family of these proteins. However, the extent to which these plant ECT (YTH family members) proteins have redundant, overlapping, complimentary or antagonistic functions has remained a mystery. This manuscript begins to address this issue by undertaking a detailed and methodical characterisation of a set of three related ECT family members as single, double and triple mutants. Important conclusions from this work are that phenotypes seen in the low m6A hypomorphs are recapitulated in the ect2 and 3, knockout mutants, and the severity of the

© 2020. Published by The Company of Biologists under the terms of the Creative Commons Attribution License (https://creativecommons.org/licenses/by/4.0/).

phenotypes increases when the ect2,3 and 4 mutations are combined. However, whilst most phenotypes are consistent with redundancy, subtle but opposite effects on direction of root growth were found, indicating for the first time that ect 2 and 3 have both redundant and specific roles in development. A detailed analysis of the effects on developmental timing and phyllotaxy and a correlation with expression patterns indicates an important role in rapidly dividing cells, particularly in root and shoot apical meristems.

Comments for the author

Although it has been reported elsewhere, it would be useful for the reader to have a tree showing the relatedness of the 13 Arabidopsis ECT family members. Possibly this could be included in the Supplemental data and referred to in the text.

Figure 5 Legend "Stimulation of root growth involves ECT4 and requires intact m6A binding sites in ECT2 and ECT3" should either be changed or additional data and text added. The requirement for the ECT2 and 3 YTH domains is clear, but an explanation and interpretation of the involvement of ECT4 is not explained in the figure, legend or the corresponding text in the Results section.

It was observed that two lines of the triple mutant complemented with the ect2 transgene had a root growth direction opposite of that seen in WT Arabidopsis, and it was suggested that this might be due to slightly higher levels of ECT2 than in WT in these lines. This is potentially quite an important finding, it would be informative if qRT-PCR could be performed to test this, or (perhaps better) if a western blot for the fluorescent proteins could be made using the complemented lines with and without the altered growth direction to test correlation with transgene product levels.

It would be informative for Figure 7A if the phyllotaxis flower angle data also included a low m6A line. Presumably this defect is restored in the complementation lines?

In the Discussion, the authors could expand further on the overlap between the phenotypes seen in the hypomorphic low m6A mutants and the those of the ect reader mutants. Are there any defects seen in the hypomprphic lines which are not seen to some level in the ect triple mutant? If not, what functions might remain for the other eight ect readers?

It would be interesting to know if any of the other ECT proteins when over expressed or under the control of ect2 promoter are able to complement the triple mutant. However, such experiments are probably beyond the scope of the current manuscript.

Rupert Fray

<u>Reviewer 2</u>

Advance summary and potential significance to field

N6-Methyl-Adenosine (m6A) has recently emerged in eukaryotes as a prevalent mRNA modification that controls many biological processes, ranging from cell differentiation, embryon development, disease controls, and responses to biotic and abiotic stresses. More recently, works from the Brodersen's team implicated the m6A-modification together with its cognate « reader » proteins (ECT2/3/4) in the control of plant development, i.e. leaf formation and trichome morphology in Arabidopsis. This manuscript is a follow-up study of this first paper, in which Arribas-Hernández is applying a large set of approaches and genetic tools to assess the impact of m6A-ECT2/3/4 module in the Arabidopsis developmental program. In summary, their data nicely show that ECT2/3/4 proteins are central to plant development and that their effects in planta can be largely explained by their ability to specifically bind the m6A modification.

Comments for the author

Arribas-Hernández et al. DEVELOP/2020/189134
«Recurrent requirement for the m6A-ECT2/ECT3/ECT4 axis in the control of cell proliferation during plant organogenesis»

© 2020. Published by The Company of Biologists under the terms of the Creative Commons Attribution License (https://creativecommons.org/licenses/by/4.0/).

N6-Methyl-Adenosine (m6A) has recently emerged in eukaryotes as a prevalent mRNA modification that controls many biological processes, ranging from cell differentiation, embryon development, disease controls, and responses to biotic and abiotic stresses. More recently, works from the Brodersen's team implicated the m6A-modification together with its cognate « reader » proteins (ECT2/3/4) in the control of plant development, i.e. leaf formation and trichome morphology in Arabidopsis. This manuscript is a follow-up study of this first paper, in which Arribas-Hernández is applying a large set of approaches and genetic tools to assess the impact of m6A-ECT2/3/4 module in the Arabidopsis developmental program. In summary, their data nicely show that ECT2/3/4 proteins are central to plant development and that their effects in planta can be largely explained by their ability to specifically bind the m6A modification.
The paper is interesting and addresses problems that are very important to the fields of plant epitranscriptomics and plant development. The experimental work is well carried out, and the conclusions drawn by the authors are supported by the presented data. The authors have also developed many genetic tools that will likely impact many areas of plant biology. Although there are still many questions remaining about the nature of the molecular targets of ECT2/3/4 readers, this work forms the basis for further work and would likely be appreciated by the readers of Development Journal. However, the specific minor points listed below have to be addressed by the authors.

Specific points.
1. In Figure 1A, the authors claim that the size of apical meristem in WT versus te234 plants is quite similar, without providing any quantitative measures. The authors should provide adequate data to support their claim.

2. The authors report that ect2 single mutant roots display a strong right slanting that is actually not observed in higher order ect mutants. Athough an opposite and compensatory effect of ect3 mutation on root slanting could be proposed to explain this observation, I wonder if the ect2 slanting phenotype could be due to a second, unlinked mutation present in the ect2 mutant background? Could the expression of WT ECT2 in ect2 mutant background rescue this phenotype?

Reviewer 3

Advance summary and potential significance to field

Authors deeply characterize the phenotypes of ect2/3/4 m6A reader loss of function mutants (Arribas-Hernández et al. Plant Cell 2018) and broadly expand the number of phenotypes published earlier. It convincingly highlights the role of m6A in plant development. The work is compatible with previous papers revealing phenotypic defects of hypomorphic mutants in m6A writers and also with a couple of those dealing with m6A erasers. They also point out that the downstream signaling cascade of m6A readers may trigger different developmental mechanisms, based on the process studied. Authors also in-depth reveal that ECT2, 3 and 4 are (in contrast to m6A writer proteins) differentially expressed, even if that was also partially revealed in their (and others') previous paper(s). They also confirm that the variants of the previously published reader constructs with the mutated binding pocket for m6A (Arribas-Hernández et al. Plant Cell 2018) are unable to rescue large part of the ect234 combinatory phenotypes presented here.
The only shortcoming of the work is that it may look for someone a bit descriptive and also slightly confirmatory. However, the amount of data presented is tremendous and the formal level of the manuscript is very high. Given the considerable interest of the community in m6A, this study may be etalon for characterizing developmental defects of mutants with defective m6A processing.

Comments for the author

Minor comments
The Introduction can be a bit abridged, as it does not go straight to the point now. I would, however, include in the discussion the comparison to animal systems – the role of m6A there and expression of readers, writers (and erasers) in development, also with regard to their developmental uniform/differential expression.

© 2020. Published by The Company of Biologists under the terms of the Creative Commons Attribution License (https://creativecommons.org/licenses/by/4.0/).

p. 4 “Many effects..” Reference to the paper(s), which demonstrated that YTH binds m6A is missing.
Abbreviations: it would be more intuitive to call mutants ect234 rather than te234 or, e.g., ect23g or ect23-2 for Gde23, the nomenclature used here is uncommon and actually pleonastic. The authors do not use such symbols in their previous paper anyway.
The line breaks in Fig 2C seem to have no reason. The graph outline is understandable but could be improved to look more understandable.
p. 12 …FLAG-ECT3W283A – Reference to their previous work is missing.
Check italicization of e.g. DR5:GFP or ECT2p:ECT2-mCherry constructs.

First revision

Author response to reviewers' comments

RESPONSE TO REVIEWERS

We thank all three reviewers for their good and constructive comments on the originally submitted manuscript. The comments have allowed us to sharpen the manuscript by correction of mistakes, shortening of some sections and expansion of others to meet a broad audience. In addition, we have strengthened the revised manuscript by inclusion of results of additional experiments as requested. Below we detail point by point how we have dealt with each of the reviewers’ comments, including explanations of the few cases in which we have taken no action to address a point raised.

Reviewer 1

1. Although it has been reported elsewhere, it would be useful for the reader to have a tree showing the relatedness of the 13 Arabidopsis ECT family members. Possibly this could be included in the Supplemental data and referred to in the text.

This has been a tough call for us. In the end, we have decided not to include a phylogenetic tree of the Arabidopsis ECT protein family for two reasons: First, as noted by the reviewer, such trees have been reported in multiple papers. Second, we have had to considerably shorten the manuscript which means that any addition of text or extra material that requires comment must be absolutely essential to make it into the revised version. We did not judge this point to meet that bar.

2. Figure 5 Legend “Stimulation of root growth involves ECT4 and requires intact m6A binding sites in ECT2 and ECT3” should either be changed or additional data and text added. The requirement for the ECT2 and 3 YTH domains is clear, but an explanation and interpretation of the involvement of ECT4 is not explained in the figure, legend or the corresponding text in the Results section.

We thank the reviewer for spotting this mistake. The legend has now been corrected.

The mistake arose from a rearrangement of figures: the right-most panels of Figure 4 (showing the growth of roots of the te234 mutant compared to those of de23) were initially placed in Figure 5, but were later moved to Figure 4 following feedback from co-authors. Although we adjusted the content of the Figure legends accordingly, we forgot to amend the title of Figure 5. Our apologies for that, and thanks again for spotting the mistake.

3. It was observed that two lines of the triple mutant complemented with the ect2 transgene had a root growth direction opposite of that seen in WT Arabidopsis, and it was suggested that this might be due to slightly higher levels of ECT2 than in WT in these lines. This is potentially quite an important finding, it would be informative if qRT-PCR could be performed to test this, or (perhaps better) if a western blot for the fluorescent proteins

© 2020. Published by The Company of Biologists under the terms of the Creative Commons Attribution License (https://creativecommons.org/licenses/by/4.0/).

could be made using the complemented lines with and without the altered growth direction to test correlation with transgene product levels.

This is a very good point, and we have tried to address it as rigorously as we could. We agree with the reviewer that the most informative analysis is measurement of protein levels. In addition to measuring protein levels in the *te234/ECT2-mCherry* (Fig. 5F), we have included data on *ect2* single mutants expressing *ECT2-mCherry* transgenes (Fig. 5A-F), and characterization of root growth in *de34* mutants (Fig. S4). These data establish that increased dosage of ECT2 is sufficient to change root directionality, because i) roots of *de34* mutants do not differ significantly from *ect3-1* and therefore left slanting in *te234/ECT2-mCherry* lines cannot be explained by the resulting genetic background after complementation, and ii) because weak overexpression of ECT2 in *ect2- 1* also causes left slanting, while knockout of ECT2, as shown in the originally submitted version, causes exaggerated right slanting. Thus, the directionality of root growth exhibits strong dependence on ECT2 dosage.

4. It would be informative for Figure 7A if the phyllotaxis flower angle data also included a low m6A line.

We have added results of analyses of two genetic backgrounds (*hakai-1* and *ABI3pro:MTA/mta-1*) with low m^{6}A content as requested. The results show that reduction of m^{6}A content causes defective phyllotaxis very similar to what is observed in *ect2/3/4* mutants. These results have been added to Figure 7 that has now been split so that the results on flower morphology and quantification of petal numbers have now been moved to new Figure 8.

Please, see additional comments below.

Presumably this defect is restored in the complementation lines?

We are quite certain that this is a true statement, based on casual observation and comparisons of complementation lines with wild type and *te234* mutants. Proving it by quantitative measurements as those reported in Figure 7 is another story, however. It is very time- and space-consuming to do these measurements with a meaningful number of plants, so although we prefer to include complementation in proof of causality between gene knockout and a particular phenotype, we have chosen to rely solely on two different allele combinations (*te234* and *Gte234*) for the phyllotaxis phenotype. We wish to emphasize that this is not a matter of laziness on our side: Every complementation test adds 4 lines to be measured (2 wild type and 2 mutant) giving a total of 8 additional lines, since both ECT2 and ECT3 would have to be analysed to be systematic. As measuring phyllotaxis of every line takes at least a full working day (30-40 plants manually measured means ~6 hours considering 10 minutes per plant), it is impossible for one person to measure 8 lines in addition to wild type and triple mutants during the window of time in which they all have mature, but still green siliques if the plants are to grow in parallel.

We do, nonetheless, agree with the reviewer that it is important to clarify whether low m^{6}A lines also display phyllotaxis defects. Phyllotaxis has not been systematically analysed in m^{6}A writer mutants, so it is valuable to know whether this is an ECT-specific defect unrelated to m^{6}A, or whether it is also seen in low m^{6}A lines. Fortunately, that fits within what we can achieve by our
standards (same growth chamber and same person to carry out the measurements on all lines used for direct comparisons). Thus, we have repeated the experiment to analyse two lines with low m^{6}A as requested.

5. In the Discussion, the authors could expand further on the overlap between the phenotypes seen in the hypomorphic low m^{6}A mutants and those of the ect reader mutants. Are there any defects seen in the hypomprphic lines which are not seen to some level in the ect triple mutant? If not, what functions might remain for the other eight ect readers?

We agree, this is indeed a very important point for discussion. We had a section in the

© 2020. Published by The Company of Biologists under the terms of the Creative Commons Attribution License (https://creativecommons.org/licenses/by/4.0/).

Discussion, "*ECT2/3/4 as mediators of m^{6}A*", in which we discussed the severity of strong loss-of-m^{6}A mutants compared to the milder *ect2/ect3/ect4* phenotypes with a focus on developmental arrest at embryo (in KOs) or very early seedling stage (strong *fip37* lines). However, we agree that the part about comparisons between the phenotypes of *ect2/3/4* and those seen in hypomorphic low m^{6}A mutants needed improvement. We have done our best make it more comprehensive and interesting in this revised version, now under the subheading "*m^{6}A-ECT2/3/4: only one of several m^{6}A-dependent regulatory axes in development*".

6. It would be interesting to know if any of the other ECT proteins when over expressed or under the control of ect2 promoter are able to complement the triple mutant. However, such experiments are probably beyond the scope of the current manuscript.

Once again, we agree with the suggestion. It is indeed an interesting experiment that we have initiated. However, due to the current length of the manuscript, we had to refrain from deeper descriptions and analyses, such as the one proposed here on redundancy, at this stage. We hope to be able to report on the results on ECT redundancy elsewhere in the near future.

Reviewer 2

1. In Figure 1A, the authors claim that the size of apical meristem in WT versus te234 plants is quite similar, without providing any quantitative measures. The authors should provide adequate data to support their claim.

We agree with the reviewer, and have now included quantitative measurements of meristem size based on the histological analyses shown in Figure 1. The results show that there is no significant difference in meristem size between wild type and *te234* mutants during the window of time analysed (Figure S1 in the supplementary material).

2. The authors report that ect2 single mutant roots display a strong right slanting that is actually not observed in higher order ect mutants. Athough an opposite and compensatory effect of ect3 mutation on root slanting could be proposed to explain this observation, I wonder if the ect2 slanting phenotype could be due to a second, unlinked mutation present in the ect2 mutant background? Could the expression of WT ECT2 in ect2 mutant background rescue this phenotype?

We consider the possibility of an unlinked mutation as the cause of the root slanting phenotype to be extremely unlikely, due to the reproducibility of the right slanting phenotype in two independent alleles of *ect2*: *ect2-1* (a SALK line) and *ect2-3* (a GABI-KAT line). Similar phenotypes observed with independently generated mutant alleles of the same genes are indeed a golden standard for proof of causality.

Nonetheless, because of the reviewer's comment, and because of the request of reviewer 1 to expand the analyses of the relation between ECT2 dosage and root slanting, we have also included phenotypic and ECT2 expression analyses of *ect2* single mutant lines expressing ECT2 transgenes (*ect2-1/ECT2-mCherry*) in Figure 5, as explained above. These lines express more ECT2 than wild type, and do indeed exhibit left slanting, strongly indicating a dosage dependence of ECT2 on root slanting.

Reviewer 3

1. The Introduction can be a bit abridged, as it does not go straight to the point now. I would, however, include in the discussion the comparison to animal systems - the role of m6A there and expression of readers, writers (and erasers) in development, also with regard to their developmental uniform/differential expression.

We thank the reviewer for this comment. We have accommodated both requests in the revised version, and agree that this improves the manuscript and make it more likely to

© 2020. Published by The Company of Biologists under the terms of the Creative Commons Attribution License (https://creativecommons.org/licenses/by/4.0/).

appeal to the broad audience of *Development*.

2. p. 4 "Many effects.." Reference to the paper(s), which demonstrated that YTH binds m6A is missing.

The references to the studies that prove m6A-binding activity of the YTH domain (Li et al., 2014b; Luo and Tong, 2014; Theler et al., 2014; Xu et al., 2014; Zhu et al., 2014) appear at the end of the next sentence, which provides the details on the molecular context responsible for this affinity and it is, therefore, a continuation of the same statement. We find unnecessary and detrimental to the readability to repeat such a long stretch of references in two consecutives sentences about the same matter.

3. Abbreviations: it would be more intuitive to call mutants ect234 rather than te234 or, e.g., ect23g or ect23-2 for Gde23, the nomenclature used here is uncommon and actually pleonastic. The authors do not use such symbols in their previous paper anyway.

We see the point, but have nonetheless kept our notation. This is not to be stubborn, and therefore requires a bit of explaining:

The abbreviations respond to the need of providing compact figures, in particular for histograms, as i) adding so much text to the figure annotations while adhering to journal policy for figure and font size is extremely challenging, and ii) it would result in figures much harder to interpret. That need was not imperative in the previous paper, which was simpler in terms of the number of allele combinations and phenotypic traits characterized. Furthermore, we believe that their use improves the readability of the text, an issue that became much accentuated in this, more complex phenotypic characterization, compared to the previous work. This system is not in conflict with the one in the previous paper. We only propose it to be used as abbreviations, well explained in a table of the manuscript. Of note, we are not confusing the literature by implying changes in gene names or allele numbers.

We have experienced that the use of "t" or "d" for triple and double mutants prevents mistakes and gives the reader the important information first. In terms of alleles, we do not wish to add -2, -3, etc. for additional combinations because for example *ect23-2* might be easily confused with *ect3-2* or misinterpreted as containing the *ect3-2* allele, which would not necessarily be the case if this system were used in a systematic manner for additional mutants. We also found that adding the G at the start helped not confusing alleles, and it comes naturally as an adjective in "the GABI double mutant" (not "the double mutant GABI").

Although we appreciate the suggestion and we see how it may seem more logic, due to the practical reasons exposed we wish to keep the notation as it is, and hope that we do not come across as stubbornly childish because of this.

4. The line breaks in Fig 2C seem to have no reason. The graph outline is understandable but could be improved to look more understandable.

We have worked a lot on that figure to make it as clear and understandable as possible. The problem is simple: differences in size at early stages, when the leaves are tiny, are barely visible in a scale that covers the entire growth range. We came up with two solutions: the classical logarithmic scale, or a double scale. After querying the opinion of several colleagues when presented with the two solutions, most chose the double scale as more intuitive and clear, and so we chose that option and wrote the reason for such double scale in the figure legend (after shortening text due to article length requirements it reads: "*A double scale in* cm^2*, indicated on the left and right sides of both graphs, is used to show in detail both early and late stages of growth*").

With respect to the outline, unless we split the two parts of the graph apart to add a new axis with numbers referring to the second scale (and we consider this option more confusing), the only solution to avoid numbers overlapping with the data points is to add

© 2020. Published by The Company of Biologists under the terms of the Creative Commons Attribution License (https://creativecommons.org/licenses/by/4.0/).

the two scales with their guides to either side of the graph (notice that we chose the horizontal guides in a way that the same number is repeated on the two scales to serve as a reference, that is 0.3 cm2 in the first pair of leaves and 1 cm2 in the second). As the guides need to meet in the point of the graph where the scale changes, we draw a vertical line as the ending point of the guides and marking the point on the horizontal axis where the scale changes. The breakage point on the vertical line serves to illustrate that there is a difference in the scales left and right of the line, and these correspond to the data curves that meet the line up and down of the breakage point. If it is only that breakage point (the crossed double line) which makes the graph confusing we could of course remove it, but we believe that it improves clarity.

5. p. 12 …FLAG-ECT3W283A – Reference to their previous work is missing.

We have now added the reference. Thanks for noticing this absence.

6. Check italicization of e.g. DR5:GFP or ECT2p:ECT2-mCherry constructs.

Again, thanks for spotting this mistake. This type of error has now been corrected.

Second decision letter

MS ID#: DEVELOP/2020/189134

MS TITLE: Recurrent requirement for the m^{6}A-ECT2/ECT3/ECT4 axis in the control of cell proliferation during plant organogenesis

AUTHORS: Laura Arribas-Hernández, Sara Simonini, Mathias Henning Hansen, Esther Botterweg, Simon Bressendorff, Yang Dong, Lars Ostergaard, and Peter Brodersen
ARTICLE TYPE: Research Article

I am happy to tell you that your manuscript has been accepted for publication in Development, pending our standard ethics checks.

Reviewer 2

Advance summary and potential significance to field

NA

Comments for the author

NA

Reviewer 3

Advance summary and potential significance to field

The study expands the repertoire of developmental defects associated with m6A readers.

Comments for the author

Authors addressed my comments or they explained that they had a different opinion. As all my specific comments were minor, I am fine with the revised version.

© 2020. Published by The Company of Biologists under the terms of the Creative Commons Attribution License (https://creativecommons.org/licenses/by/4.0/).
